# Deep learning-driven prediction of drug mechanism of action from large-scale chemical-genetic interaction profiles

**DOI:** 10.1186/s13321-022-00596-6

**Published:** 2022-03-12

**Authors:** Chengyou Liu, Andrew M. Hogan, Hunter Sturm, Mohd Wasif Khan, Md. Mohaiminul Islam, A. S. M. Zisanur Rahman, Rebecca Davis, Silvia T. Cardona, Pingzhao Hu

**Affiliations:** 1 Department of Electrical and Computer Engineering, University of Manitoba, Winnipeg, MB Canada; 2 Department of Microbiology, University of Manitoba, Winnipeg, MB Canada; 3 Department of Chemistry, University of Manitoba, Winnipeg, MB Canada; 4 Department of Biochemistry and Medical Genetics, University of Manitoba, Winnipeg, MB Canada; 5 Department of Computer Science, University of Manitoba, Winnipeg, MB Canada; 6 Department of Medical Microbiology & Infectious Diseases, University of Manitoba, Winnipeg, Canada; 7 Department of Biochemistry and Medical Genetics, University of Manitoba, Room 308, Basic Medical Sciences Building, 745 Bannatyne Avenue, Winnipeg, MB R3E 0J9 Canada

**Keywords:** Graph convolutional neural networks, Chemical-genetic interaction profiles, Gene clusters, Molecular representations, Mechanism of action

## Abstract

**Motivation:**

Chemical–genetic interaction profiling is a genetic approach that quantifies the susceptibility of a set of mutants depleted in specific gene product(s) to a set of chemical compounds. With the recent advances in artificial intelligence, chemical–genetic interaction profiles (CGIPs) can be leveraged to predict mechanism of action of compounds. This can be achieved by using machine learning, where the data from a CGIP is fed into the machine learning platform along with the chemical descriptors to develop a chemogenetically trained model. As small molecules can be considered non-structural data, graph convolutional neural networks, which can learn from the chemical structures directly, can be used to successfully predict molecular properties. Clustering analysis, on the other hand, is a critical approach to get insights into the underlying biological relationships between the gene products in the high-dimensional chemical-genetic data.

**Methods and results:**

In this study, we proposed a comprehensive framework based on the large-scale chemical-genetics dataset built in *Mycobacterium tuberculosis* for predicting CGIPs using graph-based deep learning models. Our approach is structured into three parts. First, by matching *M. tuberculosis* genes with homologous genes in *Escherichia coli* (*E. coli*) according to their gene products, we grouped the genes into clusters with distinct biological functions. Second, we employed a directed message passing neural network to predict growth inhibition against *M. tuberculosis* gene clusters using a collection of 50,000 chemicals with the profile. We compared the performance of different baseline models and implemented multi-label tasks in binary classification frameworks. Lastly, we applied the trained model to an externally curated drug set that had experimental results against *M. tuberculosis* genes to examine the effectiveness of our method. Overall, we demonstrate that our approach effectively created *M. tuberculosis* gene clusters, and the trained classifier is able to predict activity against essential *M. tuberculosis* targets with high accuracy.

**Conclusion:**

This work provides an analytical framework for modeling large-scale chemical-genetic datasets for predicting CGIPs and generating hypothesis about mechanism of action of novel drugs. In addition, this work highlights the importance of graph-based deep neural networks in drug discovery.

**Supplementary Information:**

The online version contains supplementary material available at 10.1186/s13321-022-00596-6.

## Introduction

Chemical-genetics involves the large-scale screening of compound libraries against genetically distinct cells to assess the impact of genetic differences on the activity of the drugs [1]. Chemical genetic approaches are powerful tools for generating hypotheses about the general mechanism of action (MOA) of a drug and sometimes it can help to identify the interacting target(s). The underlying principle is that changes in the dosage of a biological macromolecule (i.e., in a knockdown mutation) alter the susceptibility of the cell in response to chemicals that directly or indirectly interact with that macromolecule [2, 3]. The changes in susceptibility of cells with altered macromolecule levels, affect their abundance relative to other mutants exposed to the drug, signaling chemical-genetic interactions. The complete set of such changes, results in a characteristic chemical-genetic interaction profile (CGIP) for each drug that can then be used to characterize their MOA. While powerful in terms of rendering results, chemical-genetics is not available to the standard bioscience laboratory. The reason is that chemical-genetic approaches require large-scale genetic manipulation of cells to construct mutant libraries and robotic equipment or next-generation sequencing for high-throughput screening of the drugs against a given mutant collection.

Machine learning (ML) is an area of artificial intelligence that is commonly built from large data collections to find hidden patterns, thus providing predictive power for new data. Over the past decades, ML approaches have evolved rapidly and become a routine step in many chemical and biological applications [4]. In general, ML includes two major categories: supervised and unsupervised learning. Supervised approaches use models learned on data sets (training set) with known patterns (labels) to predict the labels of new data (test set) [5]. Unsupervised approaches work to discover the patterns existing in a given data set and classify the objects into similar groups. Supervised methods have been applied in quantitative structure property/activity relationship (QSPR/QSAR) models analysis for years in attempt to increase and streamline the rate of drug discovery [6]. An efficient ML model could filter out thousands of extraneous compounds in the virtual database and accelerate the process of finding drug candidates with the desired activity. For example, the Support Vector Machine (SVM) classification algorithm is a robust, highly accurate classification technique that is capable of handling high dimensional spaces and is widely applied in QSAR analysis [7]. Deep learning (DL) methods, which have flexible neural network architectures that allow the model to recognize distinct patterns in complex datasets, has been demonstrated to have capacities to handle various problems of drug discovery [8].

In the application of predicting molecular activities in cheminformatics, a DL model must effectively extract the underlying information from molecular structures. Traditional QSPR/QSAR models take fixed molecular descriptors, which are derived directly from the representation of molecules, as the inputs. Therefore, various molecular fingerprints generated by using different dimensions of molecular graphic information and algorithms are largely employed and fed into networks for training. For instance, Extended-Connectivity Fingerprints (also known as Morgan Fingerprints) [9], the wildly adopted topological fingerprints in QSAR models, can extract and characterize the molecular structures to enable the networks to access information about molecules. On the other hand, attempts have been made to construct models that can directly learn molecular structures while training [10–14]. Graph convolutional networks (GCNs) take molecular structures as input, aggregate low-level molecular information from atoms and bonds, and use the learned molecular representations to predict the desired properties. While the superiority of descriptor-based or graph-based representation of molecules is still debatable, the GCN-based architecture has some significant advantages over these descriptor-based models. Compared to methods that learn from fixed descriptors, GCNs operate on the molecular graph directly, enabling architectures to construct molecular representations with greater flexibility. Moreover, a number of implementations of GCNs in QSPR/QSAR tasks have shown promising results. For instance, Wu et al. [13] illustrated that the learnable featurization of molecules outperforms descriptor-based models in general. Also, Jiang et al. [15] reported that graph-based architectures can achieve superior performance on multi-task or more extensive data.

The general objective of this work was to train GCN-based models to predict CGPIs of drugs. To that end, we leveraged a large-scale chemical genetic dataset [16] in which hundreds of mutant strains of the bacterium *Mycobacterium tuberculosis* were partially depleted of essential proteins (called hypomorphs) and used to screen a compound library of 47,272 compounds. By performing gene clustering and GCN-based model training on the CGIP of *M. tuberculosis*, followed by the examination of the externally curated *M. tuberculosis* inhibitors, we show that our integrated framework is capable of identifying MOAs of chemical compounds.

## Materials and methods

### Chemical-genetic profiles—based deep learning framework

**Fig. 1:**
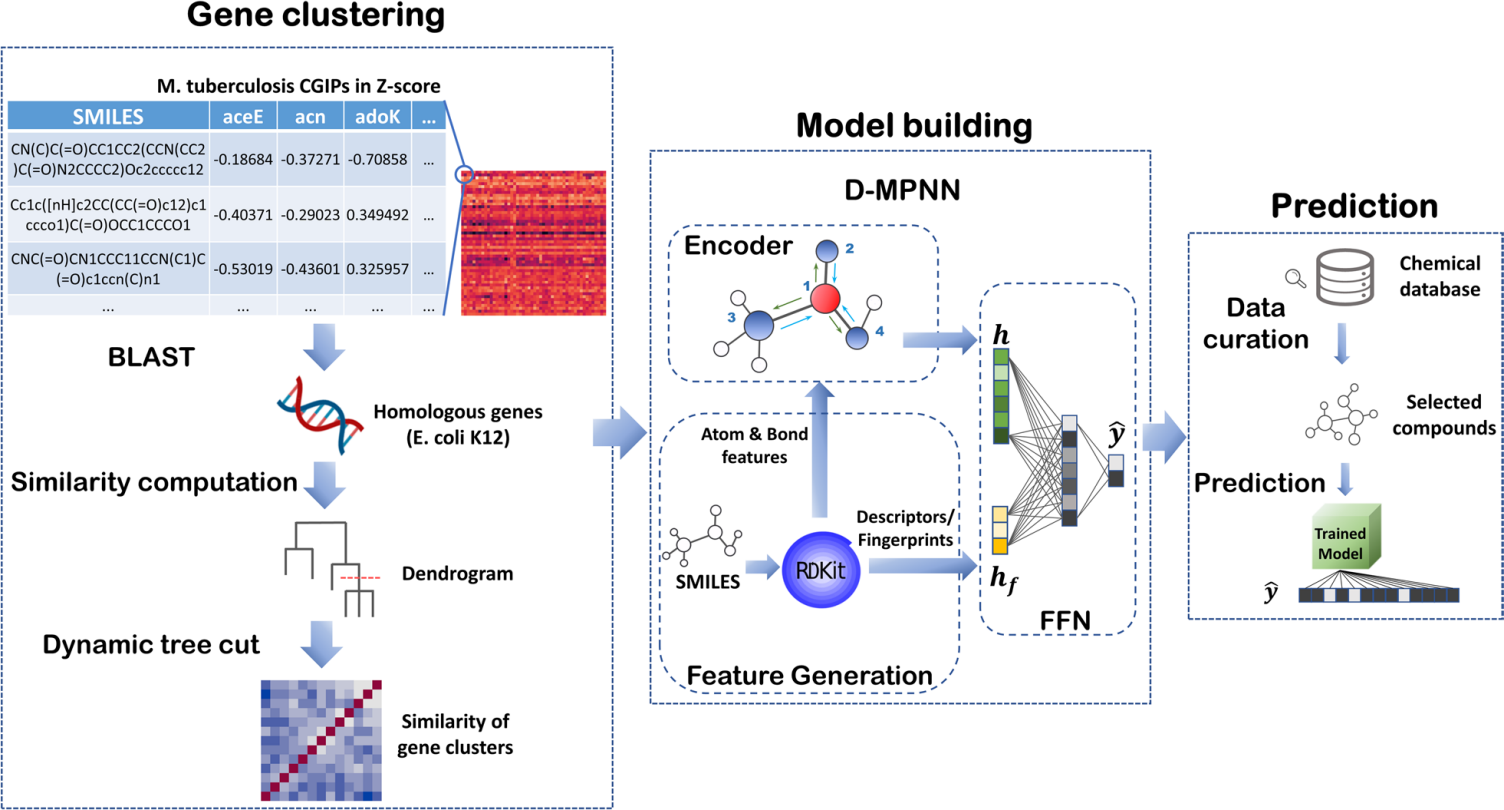
Overview of the study design. The CGIPs of *M. tuberculosis* consist of growth inhibitions (in Z-scores) of ~ 50,000 chemicals against 152 *M. tuberculosis* mutant strains (hypomorphs). The gene-level clustering was first achieved through the following processes: homology search in BLAST, gene semantic similarity computation, and cluster identification using dynamic tree cut. Using the clustered data, we trained a directed message passing network, which learned a molecular graph for each compound from the molecular features generated by RDKit. Next, we measured the performance on the test set and applied the model on several curated chemicals for further evaluation

The CGIP-based deep learning framework we propose here consists of three main steps (Fig. 1). First, we clustered the *M. tuberculosis* hypomorphs (named by the corresponding knocked down essential genes) into groups based on biological similarity of the knocked down essential gene. To facilitate clustering, we chose to use the homologous genes in *Escherichia coli* as they are more thoroughly annotated. Homology was determined with the Basic Local Alignment Search Tool (BLAST) [17], with a E-value threshold of 0.01. Then, a hierarchical dendrogram was generated according to the semantic similarity of homologs, and gene clusters were determined using dynamic tree cut [18]. For model-building, we utilized a variant of the GCNs, directed message passing neural network (D-MPNN) [14], as the architecture for training and predicting. Finally, with the trained model, we searched for externally curated compounds that had experimental results against *M. tuberculosis* strains and made predictions using them to evaluate the effectiveness of the model. Overall, this framework provides a novel paradigm of utilizing large-scale CGIPs for predicting MOAs of virtual compound libraries.

### Datasets

The CGIPs used in this study correspond to 47,217 compounds screened against 152 *M. tuberculosis* H37Rv mutant strains or hypomorphs [16]. The screen was initially developed to identify compounds that were growth-inhibitory to specific hypomorphs and hence could show potential to be developed as antimicrobial compounds against wild type *M. tuberculosis*. More than 4000 of the compounds were active against at least one of the hypomorphs, as demonstrated by their CGIPs, indicating potential for antibiotic activity against the wild type strain.

The CGIP data used in this work was downloaded from the website (https://www.chemicalgenomicsoftb.com) built by Johnson et al. [16]. The compound structures in this library are based on the simplified molecular input line entry system (SMILES), a type of specification for describing the molecular structure in ASCII strings [19]. The CGIP data includes maximum likelihood estimates of natural log fold change (LFC) of mutant strain abundance counts in each compound-strain combination compared to a dimethylsulfoxide (DMSO) solvent control. Larger absolute values of LFC indicate a larger difference in mutant strain abundance between two conditions (experimental compound vs. DMSO control). For each compound-strain combination, several types of statistical measures of the LFC are included, such as standard error of LFC, Wald test Z-score and p value [16]. Wald test Z-scores were used as a measure of strain fitness by dividing each LFC value by the respective standard error of the LFC estimate, and was assumed to follow a standard normal distribution [20]. The smaller the Z-score, the more significant is the growth inhibition of a molecule on the target. In this work, we construct our CGIPs with the usage of Z-scores measure across all the strains for every compound, as shown in Fig. 1.

### Gene clustering

Before importing the training dataset, we clustered the genes into groups based on biological similarity. This was done for two reasons: the first is that clustering reduces the total number of tasks that a machine learning model must learn and predict. Given the 152 genes in the dataset, training a multi-label algorithm with such a large number of targets is laborious and inefficient. In addition, it may be difficult to interpret the results due to the possible absence of an underlying correlation between the results. By clustering, genes with similar biological properties (i.e., gene function) can be trained on together instead of treating every gene as an individual category. Secondly, we rationalized that hypomorphs in genes from the same group may behave similarly when treated with different drugs. We used the following steps to categorize genes into clusters with biologically significant features (Fig. 1).

#### Homology search

Homology is referred to as having a common ancestor among species, and homologous genes are those which share a common ancestral sequence [21]. Genes can be clustered based on semantic comparison of biological functions contained in their gene ontology (GO) category, the standard and systematical annotation of genes and their products [22]. Bioconductor provides genome-wide annotations for 20 organisms wrapped in OrgDb, a series of R packages that store genome annotations for organisms based on mapping with entrez gene and GO identifiers [23]. However, *M. tuberculosis* is not included in the 20 organisms that Bioconductor offers. Thus, prior to computing gene similarity for *M. tuberculosis*, we first had to identify homologous genes present with the OrgDb dataset. Based on the closest phylogeny, we chose *E. coli* strain K12 [24].

The Basic local alignment search tool (BLAST) is one of the most widely used methods for sequence similarity comparisons, including DNA and protein sequences [17]. In this study, we found the *M. tuberculosis* H37Rv (RefSeq accession NC_000962.3) homologs in *E. coli* K12 (RefSeq accession NC_000913.3) using the BLAST available at the National Center for Biotechnology Information (NCBI). Protein–protein alignments were executed with expectation values (E-value) < 0.01, a threshold commonly used for inferring homology [25]. The unique protein reference sequence IDs in the BLAST result were matched with the gene symbols and an output file was generated, containing the *M. tuberculosis genes* and corresponding homologous genes in *E. coli* K12.

#### Semantic similarity computation and hierarchical clustering

Having a homologous gene list in *E. coli* K12, we used GOSemSim [26], an R package developed for semantic comparisons of GO terms, to compute similarities between the genes. Utilizing the functions in GOSemSim, the GO data associated with the biological process (BP) was introduced for measuring semantic similarity. A biological process in GO is defined as a phenomenon mediated by one or multiple gene products and marked by a change leading to a specific outcome [27]. Next, the pairwise semantic similarities were computed taking the graph-based similarity measure algorithm proposed by Wang et al. [28].

In bioinformatics, hierarchical clustering is a data mining method commonly used to detect highly correlated objects and categorize them into clusters. Objects are iteratively merged together during the hierarchical clustering process. It is an excellent method for exploratory data analysis, especially with high-dimensional data [29, 30]. In addition, it provides visualization capabilities and does not require pre-specifying the number of clusters, allowing greater flexibility in clustering tasks. We used hierarchical clustering with the average-linkage option, in which the distance between clusters is considered the average distance between all object pairs [31]. Hierarchical clustering constructs a data structure called a hierarchical clustering tree (dendrogram), where branches of the tree correspond to gene clusters. The dendrogram provides information on how objects are iteratively merged, as well as the merging height at each step.

#### Dynamic tree cut

Different types of tree cutting algorithms were proposed to determine in which cluster each object belongs according to the dendrogram [18, 32]. The most common and intuitive method of tree cutting is the fixed height branch cut. Depending on the data sets, a suitable height needs to be manually determined, and each successive branch of objects below the assigned height is perceived as a distinct cluster.

Nevertheless, the fixed height branch cut strategy does not work well in complex data structures. It is difficult to find a singular cutting height that divides all prominent branches if the dendrogram consists of nested clusters. To address this challenge, the dynamic tree cut was proposed by Langfelder et al. [18]. During the merging process of the dynamic tree cut algorithm, branches are formed from the bottom to the top of a dendrogram by applying four shape criteria: (1) satisfy the minimum number of objects; (2) low-merged objects need to be tightly connected; (3) clusters are separated by the gap between branches; (4) objects of the same branch would be excluded if they are too far away from the branch [18]. Conducting the dynamic tree cut with assigning parameters such as minimum cluster size, we can now cluster the homologs of *M. tuberculosis* H37Rv in *E. coli* K12.

#### Annotation of gene clusters

To better interpret and elucidate the biological properties of gene clusters, we assigned annotations to each cluster inferred by the biological functions of the genes contained within it. Specifically, we first matched the gene products of *M. tuberculosis* H37Rv and retrieved their protein reference sequence IDs from NCBI. Next, the reference sequences were submitted to the eggNOG-mapper v2 server [33], to assign robust functional annotations (e.g., gene name, function, GO category, Cluster of Orthologous genes (COG) category) [34]. COG categories are concise classifying annotations for proteins into one, or more, of 26 functional categories. Considering the prevailing gene functions and COG categories in the clusters, we thereby assigned a name to each *M. tuberculosis* gene cluster [35].

### Deep learning models for predicting CGIPs

#### Molecular representations

The architectures for molecular property prediction tasks are primarily categorized into three domains: deep neural networks, graph convolutional neural networks, and recurrent neural networks [36]. In general, the three approaches utilize molecular fingerprints/descriptors, molecular graphs, and textual representation of compounds respectively. Learning from various molecular representations, models can extract latent features of compounds and predict chemical properties according to different assignments.

In this work, we mainly exploit graph-based learning methods (Fig. 1). Unlike architectures that learn from the fixed featurization for entire molecules, graph-based neural networks learn and construct internal relationships between chemical properties and molecular structures directly. Applying to the QSPR/QSAR tasks, GCNs treat chemical structures like graphs, where nodes correspond to atoms and edges represent chemical bonds or other chemical interactions between them. Specifically, molecules are described as undirected graphs $G$ with node features ${x}_{v}$ and edge features ${e}_{vw}$. Since featurization of GCNs is generated in accordance with atoms and bonds, the atom/bond features are also referred to as the local features of molecules. In contrast, the molecule-level descriptors/fingerprints are denoted as global features.

#### D-MPNN architecture

Message passing neural networks (MPNNs) are categorized as variants of graph-based approaches, and the terminology was summarized by Gilmer et al. [12]. MPNNs comprise the message passing phase to iteratively aggregate local information of the molecular graph. The readout phase then constructs a global representation of the graph to make predictions on chemical properties. That is, each atomic featurization is updated by summing the information of neighboring atoms, as well as edge features. After $T$ message passing iterations, the learned hidden state across the molecule is fed into another non-linear activation to produce a single featurization for the whole molecule, which can be used for different prediction tasks. With the gradients of the loss function backpropagated through two phases, the MPNN is trained end-to-end.

Our study used a variant of MPNNs, directed message passing neural network (D-MPNN) [14], as our primary model (Fig. 1). Comparing to previously proposed MPNNs, D-MPNN considers messages associated and transferred through molecular bonds instead of atoms. Performing graph convolutions centered on bonds can avoid unnecessary loops during the message passing phase, enabling the network to build more informative and efficient molecular representations. The codes for D-MPNN was developed in Chemprop [37], which uses PyTorch [38] as the deep learning framework.

#### D-MPNN with global features

Although constructing structural information of molecules through graph encoders is prominent, it also comes with limitations compared to the networks that use fixed molecular descriptors. For instance, MPNNs suffer more to learn and extract structural information from molecules if the dataset is insufficient, which may lead to the problem of overfitting and cause poor predictions. Furthermore, since bond messages are conveyed through a molecule in $T$ iterations, the local information is hard to be passed to the entire graph. Therefore, by introducing global features such as molecular fingerprints or descriptors into the second phase of MPNN, the network can incorporate the advantages of a network trained with descriptors and improve the performance [14]. For this reason, we used 200 molecular descriptors generated by the RDKit package [39] and incorporated the features into the network by concatenating them with the graph representations at the readout phase of D-MPNN. Prior to integrating into the model, the 200 descriptors were normalized by the cumulative density functions (CDFs) to prevent the features with a broader range of values from governing the prediction.

#### Model optimization and training procedure

Two techniques frequently used for model optimizations in ML, hyperparameter optimization and ensembling, were also utilized. Bayesian optimization (BO), which was implemented in the Hyperopt package [40], was first operated to decide the optimal values of hyperparameters in D-MPNN [41]. In general, Bayesian optimization creates a probability model of the objective function and optimizes parameters by taking into account the prior information and continuously updating the current combination of hyperparameters. The four hyperparameters in D-MPNN: dropout probability, number of the message passing iterations, hidden size, and number of feed-forward layers were determined by running Bayesian optimization for 30 epochs in 20 iterations (Table 1). Ensembling, on the other hand, is realized by training the model several times with different initial weights [42]. The results of ensembling were averaged together, aiming to find an optimal and robust prediction.

**Table 1:** Bayesian optimization for hyperparameters in D-MPNN with RDKit descriptors

Hyperparameters	Values
Dropout probability	[0, 0.4] (Interval: 0.05)
Number of message-passing iterations	2, 3, 4, 5, 6
Number of feed-forward layers	1, 2, 3
Hidden size of D-MPNN	[300, 2400] (Interval: 100)

We adopted the data splitting strategy where a whole dataset is divided into three subsets (training set, validation set, and test set) following the ratio of 80:10:10. Normally, samples are randomly distributed in the three subsets following specific ratio (random splitting). In this study, we used a scaffold splitting [13], which imposes the subsets of data to share less molecular scaffolds. In particular, during the splitting process, RDKit [39] calculates a Murcko scaffold score for every molecule and they are partitioned into three subsets based on the scaffold scores. By doing so, compounds between bins share little molecular scaffolds, and thus fulfilled the scaffold diversity and represent a more challenging and realistic context for model evaluation [43].

After gene clustering, each molecule corresponds to multiple clusters and the prediction of each gene group is mutually independent, thus forming a multi-label task. The last layer of the model was set to a vector with the number of tasks, corresponds to gene clusters. As our goal was to predict the growth inhibitory activity of molecules in *M. tuberculosis* clustered hypomorphs, we treated the multi-label task as a binary classification by applying the sigmoid function to the last layer of the model and trained with binary cross-entropy loss. With the optimized hyperparameters, five models with different initial weights were trained and ensembled to generate the prediction with more robustness. The output of each unit varies from 0 to 1, which can be interpreted as the probability of growth inhibitory activity occurring in each hypomorph cluster.

#### Model evaluation strategies and metrics

Label based metrics were used for the multi-label evaluation where the metrics for each subset were computed individually and they were macro-averaged to obtain the overall results. We consider the area under curve (AUC) of receiver operating characteristic (ROC) curve, abbreviated as AUROC, as our primary performance metric for classification tasks. ROC is a comprehensive indicator of continuous variables of sensitivity and specificity, it can be viewed as an infinite number of points, and each of these points represents a classifier. Given the prediction of consecutive scores, we binarized the result in the decision-making process to derive the activity of compounds against each gene cluster. In binary classification, the most commonly used threshold for identifying classes is 0.5. In this work, because of the class imbalance in our data, we performed threshold-tuning using Youden’s J statistic [44]. Specifically, based on the ROC curve for each cluster on the validation set, the optimum cutoff falls on the location where the sensitivity plus specificity minus one is maximized. Besides AUROC, one of the most commonly used metrics for classification with imbalanced data, the area under the curve of the precision-recall cure (AUPRC) was also presented. Having the determined thresholds for each cluster, accuracy and F1 score were calculated.

#### Baseline models

We compared the D-MPNN with RDKit descriptors with five baseline models under different combinations of DL architectures and molecular features. The baseline architectures include D-MPNN, generic MPNN where atom/bond messages are conveyed on the undirected graphs, and feed-forward neural network (FFN). The calculated global features include RDKit descriptors and binary Morgan fingerprints. For graph-based models, we also presented the results of training without the global features. Specifically, there are six model settings trained and evaluated in this study: (1) D-MPNN with RDKit descriptors; (2) D-MPNN; (3) MPNN with RDKit descriptors; (4) MPNN; (5) FFN with RDKit descriptors. (6) FFN with binary Morgan fingerprints. For consistency, the hyperparameters of all models were optimized by Bayesian optimization on the scaffold split. All models were trained with an ensembling size of five.

## Results

### Gene clustering analysis

After performing the homology search between *M. tuberculosis* and *E. coli* through BLAST and the protein sequence alignment with E-value < 0.01, we obtained 122 homologous genes in *E. coli* strain K12 that correspond to the genes in *M. tuberculosis*. Additional file 2: Table S1 provides the complete list of 122 available genes for both organisms, including their products represented in the protein reference sequence. Using functions wrapped in the package GOSemSim [26], the GO data was prepared with biological process. The semantic similarities among GO terms were then calculated with the graph-based similarity measurement proposed by Wang et al. [28]. After performing all the preceding procedures, we obtained 107 *E. coli* K12 homologs remained for clustering.

**Fig. 2:**
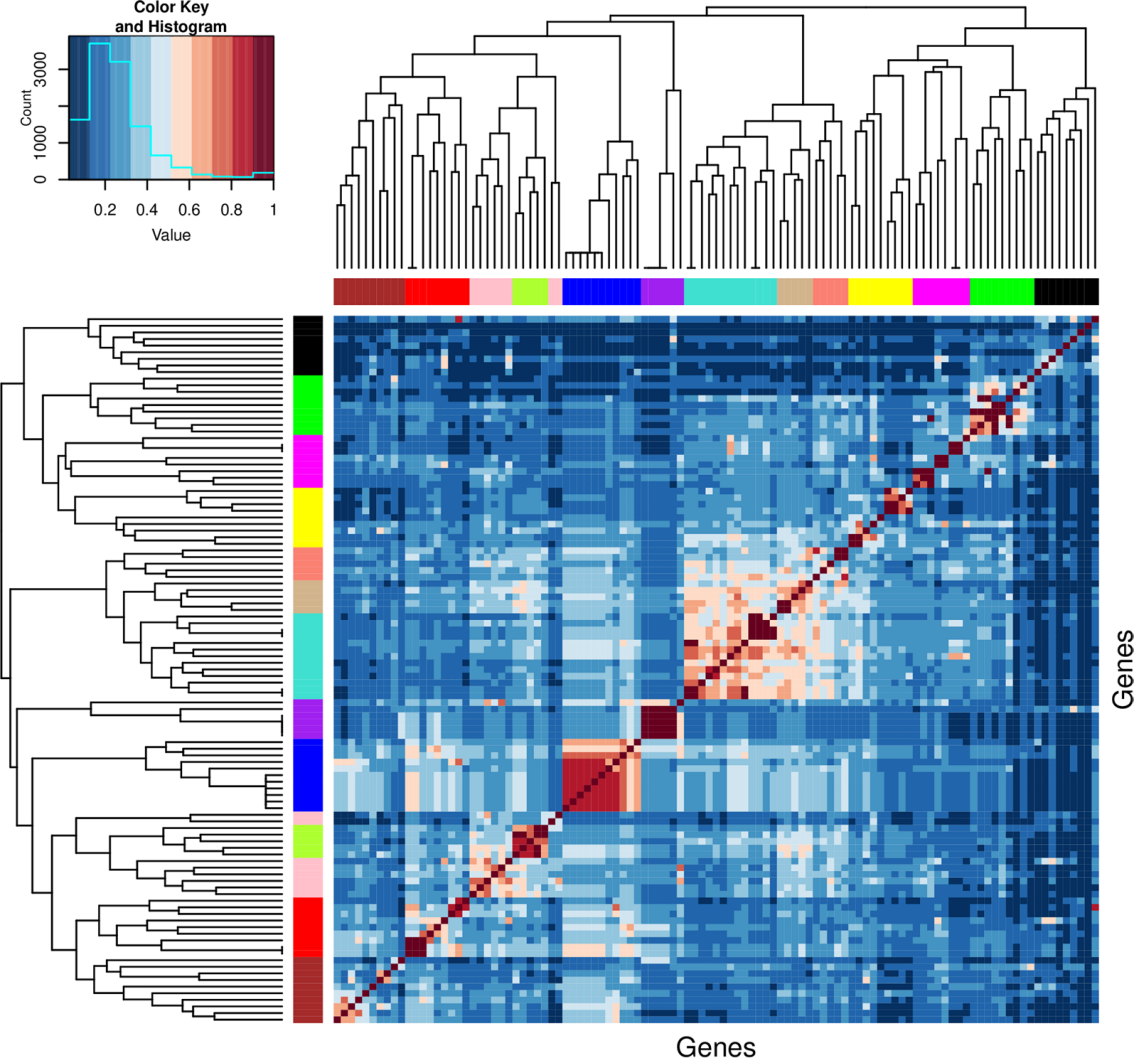
Clustering result of homologous genes in *E. coli* K12 displays in a heatmap, dendrograms, and color bars. The heatmap and dendrogram were generated by hierarchical clustering with average linkage. The heatmap is symmetrical about the diagonal, the more similar two homologs are, the closer the value between them is to 1 (red). The two dendrograms and their color bars on the top and left sides of the heatmap are identical. The vertical distances on each branch of the dendrogram indicate the relatedness of genes. Applying the dynamic tree cut algorithm on the tree, the clusters were formed as shown in the color bars below the dendrograms. The 13 gene clusters are represented in different modules in the color bar

Based on the semantic similarity matrix of homologs, the hierarchical clustering with the average-linkage was performed using the hclust function [45]. It constructed a hierarchical clustering tree containing 107 homologous genes. We applied the dynamic tree cut algorithm to determine the gene clusters based on the dendrogram and the dissimilarity matrix. Figure 2 shows the heatmap and dendrograms of the homologous genes in the *E. coli* K12 strain, together with the result of the dynamic tree cut represented in the color bars below the dendrograms. In the dynamic tree cut algorithm, we limited the minimum cluster size to 5 and enabled the parameter partitioning around medoids (PAM) to assign more outlying objects into clusters. Thus, we clustered the genes into 13 groups and each module color in the color bars shows cluster membership as determined by the dynamic tree cut algorithm. Genes with closer similarity values formed checkerboard-style blocks in the heatmap and they were grouped into the same gene cluster by the dynamic tree cut approach.

Subsequent to clustering, we applied the same graph-based similarity measurement proposed by Wang et al. [28] to calculate the pairwise semantic similarity scores among the 13 gene clusters. Additional file 1: Fig. S1 visualizes the pairwise similarity matrix of generated results. The matrix is diagonally symmetrical, the more similar two gene clusters are, the greater the value between them. According to the similarity matrix, most gene clusters have similarity scores ranging from 0.2 to 0.5. Cluster 4 and cluster 5 have the least similarity score between them (0.13), while the highest cluster-level similarity is found between clusters 4 and cluster 7 (0.59).

Given the clustering results based on the homologous genes in *E. coli*, we then substituted the homologs to the corresponding *M. tuberculosis* genes and formed the clustering results containing 13 distinct *M. tuberculosis* gene groups. Notably, during the homology search in BLAST, the *M. tuberculosis* genes *fadD30* and *fadD32* aligned to the same homologous gene *fadD* in *E. coli*. Similarly, *M. tuberculosis kasA* and *kasB* showed homology to the *E. coli fabF*. Therefore, although 107 homologs exist in the dendrogram, a final clustering result consisting of 109 M*. tuberculosis* genes was obtained.

Taking the protein sequences retrieved from NCBI as input, the eggNOG-mapper [33] generated annotation for genes and their COG categories. We then gave annotations for clusters according to their biological properties inferred by gene functions in them. Table 2 lists the given cluster names, the number of gene members, and the COG categories for each cluster. The complete summary of *M. tuberculosis* gene functions can be found in Additional file 3: Table S2, which includes additional information on gene names, protein reference sequence ID, and gene functions.

**Table 2:** Cluster name, number of genes, and COG categories for *M. Tuberculosis* gene clusters

Clusters (C)	Cluster name	# Gene	COG categories
C1	Tricarboxylic acid cycle and central carbon metabolism	9	O; C; E; F
C2	Translation and tRNA synthesis	11	M; J
C3	Non-aromatic amino acid biosynthesis	13	E; H
C4	Aromatic amino acid biosynthesis	5	E; H
C5	Stress protection and pathway equilibration	9	D; O; T; U; E; G; P
C6	tRNA and nascent polypeptide modification	10	M; T; J; E; F; S
C7	Folate and purine metabolism	5	C; E; F; H
C8	DNA replication and transcription regulation	9	K; L; H; I; O
C9	Lipid and lipid cofactor synthesis	11	F; H; I; Q
C10	Glycolysis and gluconeogenesis	8	C; E; F; G
C11	Peptidoglycan and precursor synthesis	6	D; M; G
C12	Purine and pyrimidine metabolism	8	M; F; G
C13	Porphyrin-compound synthesis	5	H

### D-MPNN for classifying chemical compounds

#### Data preparation

As stated in the previous section, the Wald test Z-score of LFC measures the magnitude of growth inhibitory activity for each compound against *M. tuberculosis* hypomorphs. In order to perform binary classification on the activities of the molecules, a threshold of Z-score is required to assign binary labels for each compound in every *M. tuberculosis* gene cluster. Deciding an appropriate cutoff for Z-score is critical, as the accuracy of predictions would be affected if compounds were added to the categories that share little traits with other compounds. Having several experiments on different Z-score thresholds in an unsupervised way, we decided to use − 4 as the class criteria. Specifically, a Z-score less than or equal to − 4 was considered to have an inhibitory effect and labeled with 1. In contrast, a Z-score greater than − 4 was marked with 0, indicating the chemical has no activity toward the target.

To summarize, calculating molecular scaffolds and splitting the dataset with the subsets ratio, 47,217 compounds from Johnson et al. [16] were divided into a training set (37,773 compounds), a validation set (4721 compounds), and a test set (4723 compounds). In order to perform the classification task on the molecules after clustering the genes, it was necessary to create the cluster-level targets for each molecule. To achieve this, for each cluster, we took the median of Z-scores that were contained in the cluster as the cluster-level targets. Additional file 1: Fig. S2 shows the distribution of Z-score in each cluster in the form of kernel density estimate plot. Next, we applied Z-score = − 4 as the class criterion to binarize labels for classification. Table 3 lists the number of positive labels in each cluster for the three subsets. It is clear that the positive labels for each cluster had also been divided into the data bins in proportion to the ratio (80:10:10) after the scaffold splitting. For the whole data, the average percentage of positive labels across clusters is 4%.

**Table 3:** Number of active molecules in the scaffold split after binarization of labels

Clusters (C)	C1	C2	C3	C4	C5	C6	C7	C8	C9	C10	C11	C12	C13
# Active in training set	1246	994	1281	1583	1542	1322	1554	1509	1256	1258	2373	1290	1968
# Active in validation set	168	140	174	216	209	185	218	195	168	163	287	183	258
# Active in test set	153	133	158	216	182	182	207	196	178	164	279	184	300
# Active in total	1567	1267	1613	2015	1933	1689	1979	1900	1602	1585	2939	1657	2526

#### Model performance of D-MPNN classifier

**Fig. 3:**
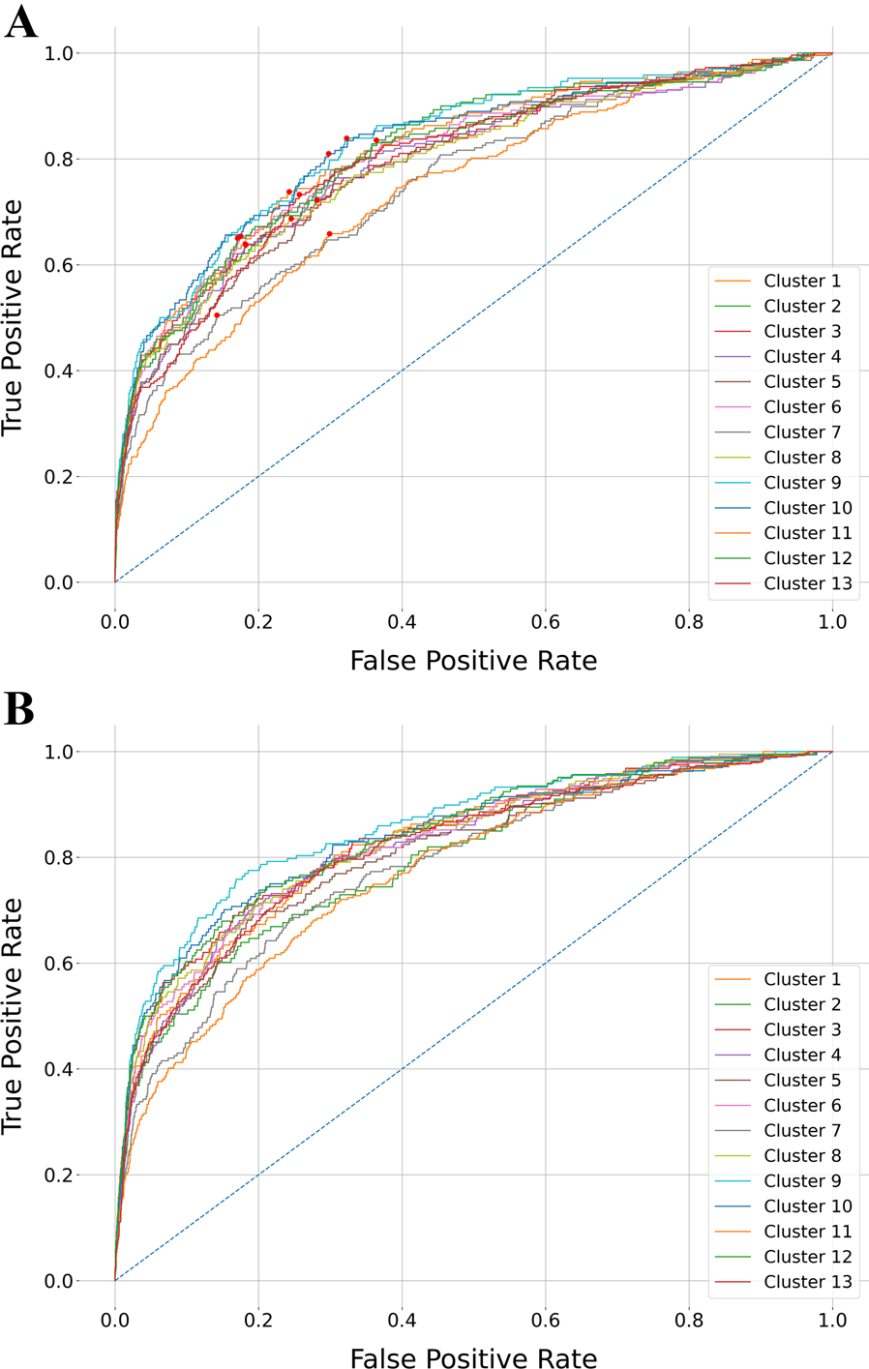
Receiver operator characteristic curves from the predictions of D-MPNN with RDKit descriptors. **A** Validation set. **B** Test set. The red dots in **A** represent the optimum cutoffs determined by Youden’s J statistic on the validation set

To evaluate the performance of our models, we drew the ROC curves of the validation set as shown in Fig. 3A, in which ROC curves of 13 clusters were plotted in the exact coordinates. The averaged AUROC score across 13 clusters achieved 0.80 on the validation set. The red dots on the ROC curves denote the position of the optimum cutoffs as identified by Youden’s J statistic [44]. To keep a fair and consistent comparation in predicted results, the cutoffs for the test set and the curated compounds were also determined by the cutoffs calculated in the validation set. The average threshold across clusters is 0.05. Comparing to the default cutoff 0.5, the optimized thresholds are more lenient and thus the model tends to classify more positive classes after the threshold-adjusting. The complete summary of cutoff for each cluster can be found in Additional file 1: Table S3. The ROC curves of gene clusters measured on the test set are presented in Fig. 3B, in which the averaged AUROC is 0.82.

**Fig. 4:**
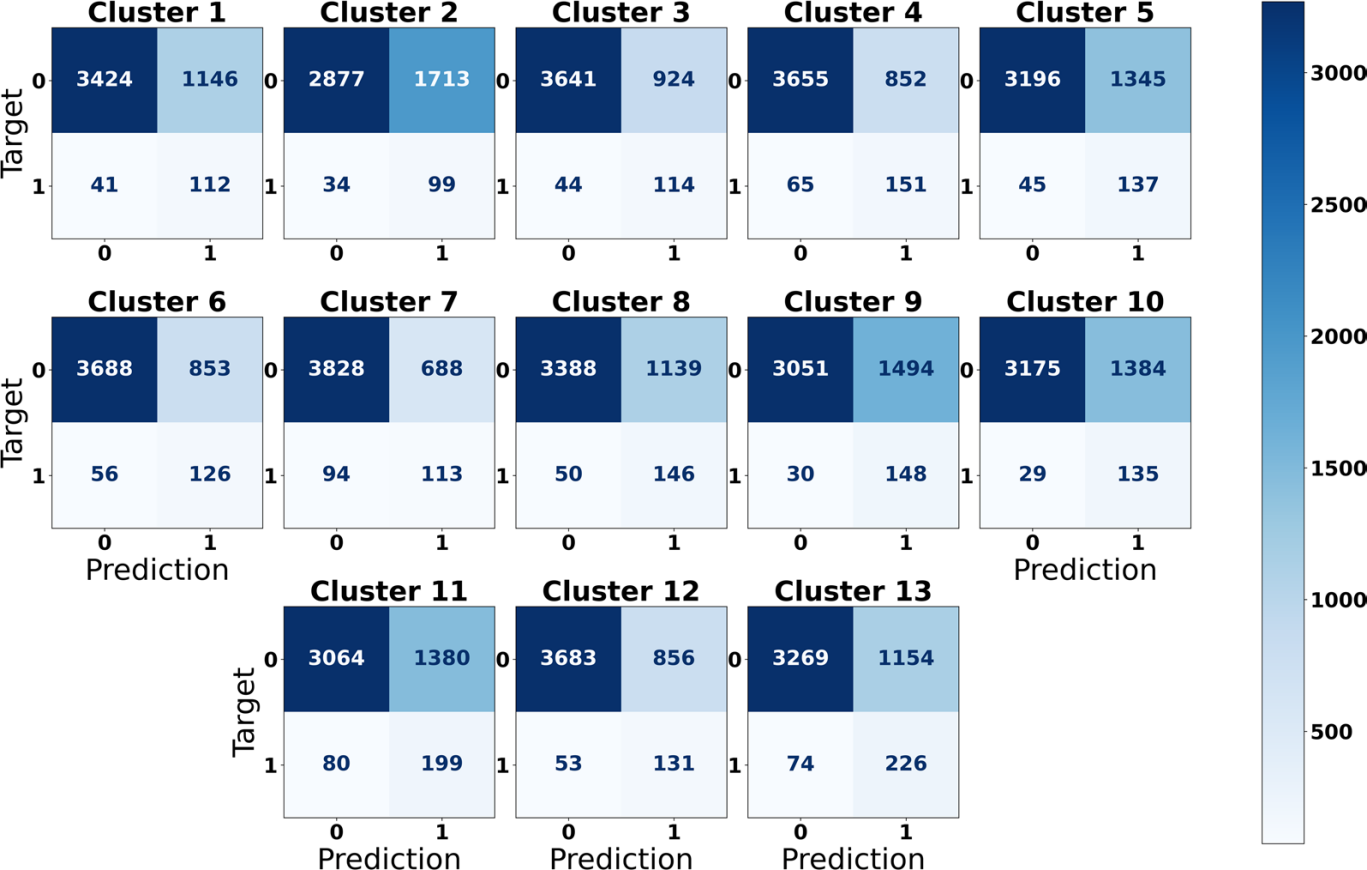
Confusion matrices of the predictions of D-MPNN with RDKit descriptors for each gene cluster on the test set. In each matrix, top left results for true negatives (TN), top right results for false positives (FP), bottom left results for false negatives (FN), and bottom right results for true positives (TP)

We also evaluated the performance of the multi-label classifier in the form of confusion matrices after binarizing the prediction of the test set using the optimum cutoffs acquired from the validation set (Fig. 4). The confusion matrix for binary classification consists of four entries, where top left results for true negatives (TN), top right results for false positives (FP), bottom left results for false negatives (FN), and bottom right results for true positives (TP). Similar to the imbalanced classes distribution in the data, here we observed that the classifier also generated more results in the inactive class (73%) than the active (27%). Regarding the accuracy, the average accuracy of thirteen clusters is 0.75, of which cluster 7 achieving the highest (0.83) and cluster 2 the lowest (0.63).

### Performance of baseline models of classification

**Fig. 5:**
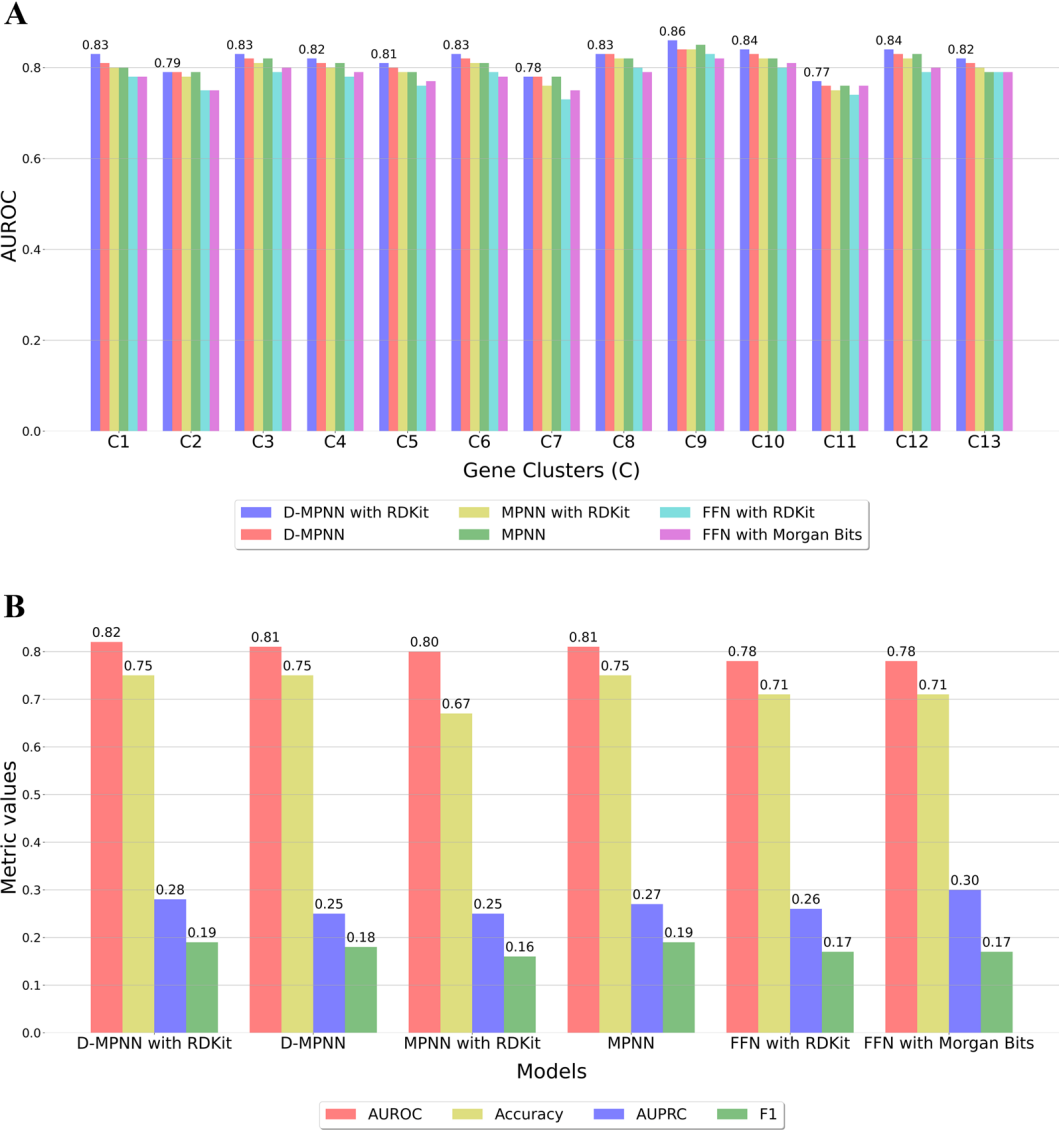
Classification metrics for the D-MPNN with RDKit descriptors and baseline models on the test set. **A** AUROC scores in each cluster for all models, only the values of the D-MPNN with RDKit descriptors are displayed. **B** The averaged metrics (AUROC, accuracy, AUPRC, F1) over clusters for the D-MPNN with RDKit descriptors and other baseline classifiers

We first compared the performance of the D-MPNN with RDKit descriptor to the five baseline models on the test set of the scaffold split. The AUROC scores for all classification models in each individual cluster are shown in Fig. 5A, where only the values for the D-MPNN with RDKit descriptors are displayed. Regardless of whether additional molecular descriptors were concatenated, the D-MPNN classifiers topped in the overall performance in terms of AUROC. With the assistance of RDKit descriptors, the D-MPNN classifier achieved the highest scores in all clusters. In general, the MPNN classifiers are the second-best right next to the D-MPNN among clusters, except for cluster 9 and cluster 11. From Fig. 5A, it is obvious that all six models had the most difficulties in the classification tasks for cluster 7 and cluster 11. Figure 5B shows the averaged AUROC, AUPRC, accuracy, and F1 score over all clusters. the D-MPNN with RDKit still outperformed or on par with baselines according to the value of AUROC (0.82), accuracy (0.75), and F1 (0.19). However, the best AUPRC (0.30) occurred in the FFN trained by binary Morgan fingerprints. The two FFN models trained with different global features achieved very close results, acquiring the same AUROC (0.78) and F1 (0.17). The performance details for all clusters and models can be found in Additional file 1: Table S3.

Besides evaluating the models on the data subsets partitioned by the scaffold splitting, we further adopted a ten-fold cross-validation (CV) approach with random splitting to decrease the noise in the results for comparison purposes (Additional file 1: Fig. S3, Table S4). The thresholds of gene clusters were aligned with the values obtained from the validation set of the scaffold split. The performance results were averaged over the ten-fold and the error bound for a population mean (EBM) at the 95% confidence level (Student’s t-distribution) were calculated. In high agreement with the previous results, the D-MPNN with RDKit descriptors has consistently exhibited the best performance in all clusters according to AUROC (Additional file 1: Fig. S3A). Regarding the effect of adding the 200 RDKit descriptors, while the performance improvement of the D-MPNN was observed in every gene cluster, the MPNN yielded slightly inferior performance with the incorporation of the features. For the averaged performance over all clusters and CV folds (Additional file 1: Fig. S3B), the D-MPNN with RDKit descriptors achieved the highest AUROC (0.79 $\pm$ 0.01), AUPRC (0.29 $\pm$ 0.02), and F1 (0.19 $\pm$ 0.01). On the whole, with the exception of the accuracy of the FFN trained on the binary Morgan fingerprints, the EBM at the 95% confidence level of metrics were all constrained within 0.02, indicating the results of different folds were well converged and reasonable comparisons can be made.

## Discussion

Accurate prediction of the potential bioactivity along with the potential target information would empower informed compound selection and prioritization, eliminating the need for expensive and laborious high throughput screening of compound libraries. Here, we developed a well-integrated approach for predicting MOA with the use of the large-scale *M. tuberculosis* CGIPs [16]. The clustering employed the gene ontologies of annotated homologs in *E. coli* K12 and a graph-based model was trained in an end-to-end manner to predict the cluster-level growth inhibitory activities from the learned molecular representations. Due to the lack of a ground truth for unsupervised clustering, we calculated the pairwise semantic similarity scores of clusters using the method proposed by Wang et al. [28] and inferred their biological functions of the genes within the clusters, in an attempt to validate the results and interpret the biological meanings behind the clusters. Although it is a more intuitive and simpler way to classify the *M. tuberculosis* genes based on the Wald test Z-scores of the LFC estimate directly, we carried out clustering without involving the CGIPs for two reasons. First, we intended to leverage the external knowledges to yield biologically meaningful gene classes. Second, since we would train growth inhibition prediction models on the Z-score, repeated use of these values in two phases would amplify the impact of possible experimental artifacts from the data.

In our experiments, the complexity of models was primarily determined by the hidden size and the number of feed-forward layers, which were both optimized by Bayesian optimization. Additional file 1: Table S5 lists the summary of hyperparameters and the number of model parameters for the compared models. Among all models, the FFN with RDKit has the fewest number of parameters (~ 0.3 M) while the MPNN with RDKit has the largest number of parameters (~ 5.7 M). Nevertheless, the performance advantages of the MPNNs do not necessarily come at the expense of models’ complexity. For example, compared to the FFN with Morgan Bits that has about 2.3 M parameters, the baseline model D-MPNN contains fewer number of parameters (~ 1.5 M), but it consistently outperformed over all other baselines in terms of AUROC. The major advantage of the MPNNs over the traditional DL architectures such as FFN is that they can efficiently extract relevant representation of molecules tailored to the desired properties. For instance, topologically adjacent atoms are more likely to interact with each other and in some cases can form functional groups. This type of information could be potentially reflected on the initial atom and bond features. By iteratively aggregating local information of molecules during the molecular representation learning process, the MPNNs are able to encode all substructures with greater flexibility, and thus form global representation that is essential to the target predictions.

According to the evaluation on the test set of the scaffold split and the ten-fold CV, the D-MPNN with RDKit classifier achieved greater or comparable performance for every cluster compared to baseline models according to all computed metrics. It showed the strengths of learning molecular representations through graphs with directed messages, indicating that the model has learned to associate chemical properties to growth inhibition of particular clustered hypomorphs, which relates to the MOA of the compounds. Despite the average accuracy of clusters on the scaffold-based test set was 0.75 after we binarized the predictions, it is worth stating that the average accuracy could reach to 0.96 if we use 0.5 as the thresholds. Still, we chose more lenient cutoffs to reduce the number of false negatives in the prediction and minimize the effect of the skewed class distribution of data albeit with relatively poorer results in terms of accuracy and precision.

**Fig. 6:**
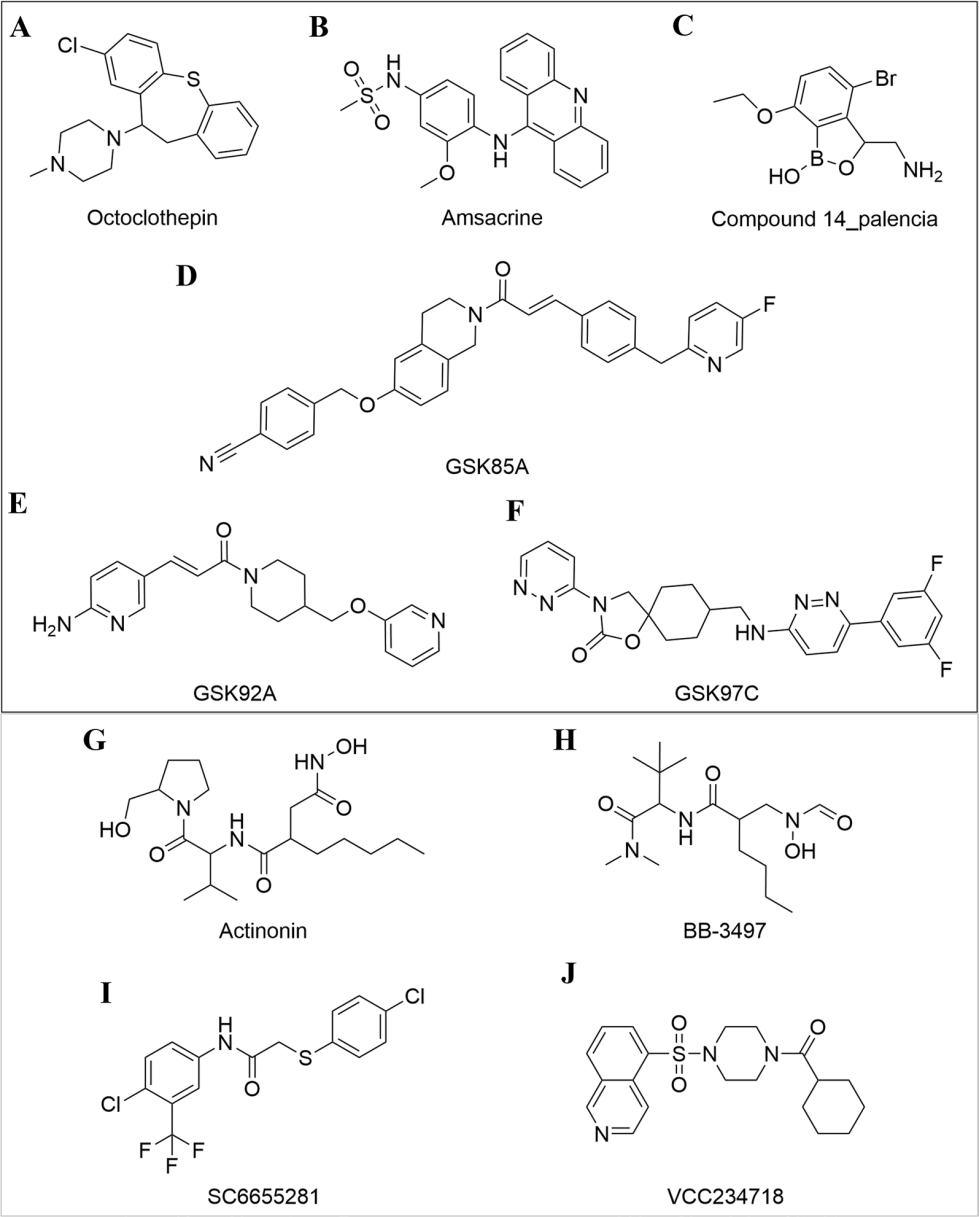
The prediction results of the example drugs. Drugs that were correctly predicted by the D-MPNN with RDKit descriptors classifier as growth inhibitors of hypomorphs within the target gene groups (Top black box). Name and structure of compounds with false-negative predictions (Bottom grey box). **A** Octoclothepin is an ATPase inhibitor of ParA in Cluster 5. **B** Amsacrine is an inhibitor of TopA in Cluster 8. **C** Compound 14_palencia is an inhibitor of LeuS of in Cluster 2 (**D**–**F**) Three AspS (in Cluster 2) inhibitors (GSK85A, GSK92A, GSK97C) were predicted to be active in all gene groups. **G**, **H** Actinonin and BB-3497 are two inhibitors of Def in Cluster 6. (I, J) SC-6655281 and VCC234718 inhibit GuaB2 in cluster 12

**Fig. 7:**
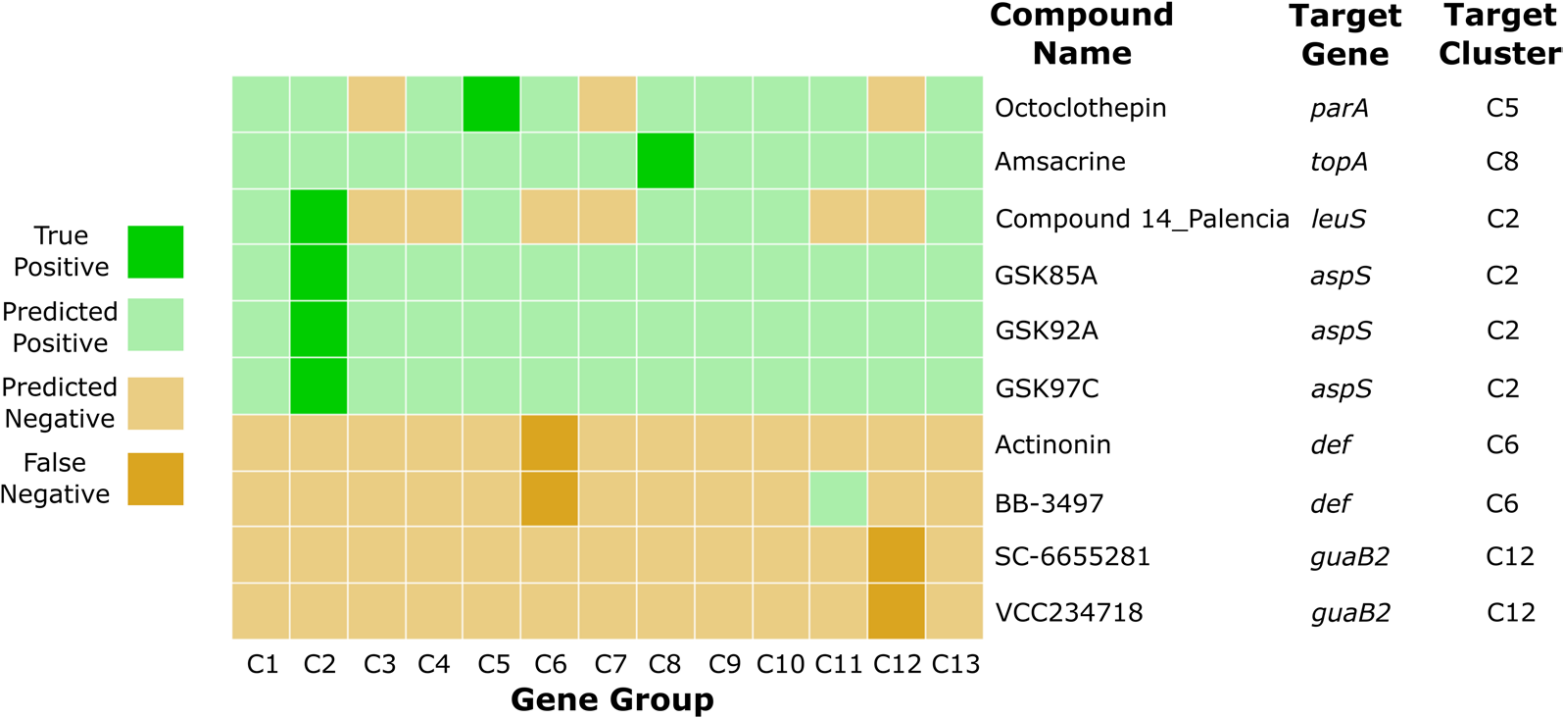
Results of ten examples of *M. tuberculosis* inhibitors predicted by the D-MPNN with RDKit descriptors. Applying the cutoffs derived from ROC curves of the validation set, the predictions were binarized. In the heatmap, light green represents a positive prediction and light yellow represents a negative prediction. If the positive result predicted by the model matches the ground truth, the color is shown as dark green (TP), whereas if the result does not coincide with the label, the color appears dark yellow (FN)

To investigate the effectiveness of our method with compounds outside of the Johnson et al. data [16], we evaluated additional drugs curated from the literature (Additional file 4: Table S6). These compounds were selected based on (1) whole-cell inhibitory activity against wild-type *M. tuberculosis* or *M. smegmatis* and (2) biochemical validation of the molecular target. For each compound, we assigned positive labels (1) to the cluster in which the *M. tuberculosis* target-coding gene was within that cluster. This assignment was based on the hypothetical scenario that the drug affects the susceptibility of the hypomorph with depletion of the target essential protein. Next, we generated the prediction for the compounds by the trained D-MPNN classifier. Applying the cutoffs derived from ROC curves of the validation set, we finally assigned binary labels to the prediction. In a multi-label classification scenario, a classifier could take or generate more than one positive label for each sample. Nevertheless, because the complete chemical-genetic profiles of these selected inhibitors are unknown, we were only able to assign true-positivity, as the growth inhibitory activity against the other hypomorphs remains unknown. Here, we investigate ten examples of such *M. tuberculosis* inhibitors (Fig. 6), including correct and incorrect predictions. To better visualize the cluster-level labels and results, we show the predicted results of ten *M. tuberculosis* inhibitors predicted by the D-MPNN classifier in a form of heatmap with a four colors scheme (Fig. 7).

Figure 6A–F displays the structures of the six drugs for which the growth inhibitory activities against specific genes were correctly identified by the D-MPNN classifier. These predictions are shown in the first six rows of the heatmap (Fig. 7). Octoclothepin (Fig. 6A) is an antipsychotic drug that has been found to function as an ATPase inhibitor of ParA, a chromosome partitioning protein in the *M. tuberculosis* [46]. ParA interacts with the DivIVA complex and cell wall metabolism, demonstrating high order linkages in the cell cycle [47]. Among all clusters, the model predicted octoclothepin to exhibit activity in ten groups, including Cluster 5, where *parA* is located. Similarly, amsacrine (Fig. 6B) was predicted to be active against all gene clusters, despite the fact that *topA* (in cluster 8) is the only known in vitro target in *M. tuberculosis* [48]. DNA replication is a central process that contributes to cell cycle regulation, among other factors [49]; therefore, we infer this might be the reason that the model predicted octoclothepin and amsacrine as active against multiple groups in addition to their ground truth targets

The genes *leuS* and *aspS* are in Cluster 2 and both belong to the same COG category (Translation, J). Compound 14_palencia (Fig. 6C) inhibits LeuS by forming an adduct with AMP and together binding the ATPase pocket [50]. The positive labels of prediction for this compound not only appeared in Cluster 2, but also in the other six clusters (Cluster 1, 5, 8, 9, 10, 13). On the basis of the properties of the compound 14_palencia and the predictions of the model, we hypothesize that the drug might also antagonize ATPases in other classes, such as GyrA (Cluster 8), GyrB (Cluster 8), ParA (Cluster 5), MenE (Cluster 9). Three AspS inhibitors GSK85A, GSK92A, GSK97C (Fig. 6D–F) [51] were predicted to be active in all gene groups. The three inhibitors have little in common besides being linear, highly flexible, and highly functionalized, which may contribute to their predicted activity against all gene groups.

Among the curated compounds we collected, we also present four examples of false negative predictions (known in vitro activity, but predicted to be inactive) (Fig. 6G–J, Fig. 7). The compounds actinonin and BB-3497 are known to inhibit *M. tuberculosis* Def in Cluster 6 (Fig. 6G, H) [52]. Nevertheless, the model missed the ground truth for these compounds. Moreover, they were not predicted as active against any other gene groups (except Cluster 11 for BB-3497). The two drugs share some similarities as they are both small and peptide-like. Additionally, both SC-6655281 and VCC234718 inhibit *M. tuberculosis* GuaB2 in Cluster 12 (Fig. 6I, J) [53, 54]. However, the D-MPNN classifier predicted that neither molecule had activity within any gene cluster. Just as any other false negative predictions, this phenomenon is very likely due to the fact that there is the absence of this type of compounds in our training set or the model itself failed to learn enough information to make the correct prediction.

We note that due to the extremely imbalanced distribution of classes, we encountered similar difficulties in terms of precision for both test set and the curated drugs. Data imbalance is a common issue in ML, particularly for drug discovery, where models inevitably learn more characteristics of majority class as well as a greater tendency to predict more false positives when negative samples dominate positives. A great amount of efforts have been devoted to address the problem, but the existing methods still have shortcomings and the problem has yet to be properly tackled [55]. On the other hand, an appropriate threshold can also largely affect the prediction results and model performance after binarization. However, in the realistic practice of finding potential drugs by ML models, it is usually unnecessary to binarize the predictions of the classifier. In our case, the results of a binary classifier are continuous values that can be interpreted as probabilities, and by ranking the predicted scores, we can select the most promising compounds for further experiments.

To summarize, a multi-label D-MPNN classifier was trained on a set of gene clusters of *M. tuberculosis* which achieved an excellent performance and outperformed other baseline models. Besides the subsets from the data, we examined the D-MPNN classifier on multiple *M. tuberculosis* gene inhibitors from the existing literature and explored the possible explanations behind the correct and incorrect predictions of the ten compounds. Since an outcome of a QSAR/QSPR model could be influenced by various factors, such as the diversity of compounds in data, the capabilities and performance of a ML model, or the choice of threshold in the phase of the decision making, it is hard to interpret the latent logic of a ML model rather than just simply accept the back-box results. However, it is encouragingly possible that both correct or incorrect predictions could become an excellent opportunity to investigate the MOA of compounds and even use for drug repurposing. Therefore, it remains necessary to make more efforts to improve not only the capability of forecasting, but also the interpretation of the model for future research of GCNs in the drug discovery field.

## Conclusion

In this study, we provided a comprehensive workflow for predicting chemical genetic profiles of drugs in *M. tuberculosis*. The clustering method first matched genes of *M. tuberculosis* with those of *E. coli* K12 by performing protein–protein alignments. Then, we calculated the semantic gene similarity of biological process for the homologs and performed hierarchical clustering. Utilizing the clustered data, a D-MPNN architecture was employed and a prediction of growth inhibitory activity in each gene group was made. With the performance of the trained network and predicted results of the curated compounds, we demonstrate that our approach effectively created *M. tuberculosis* gene clusters, and the trained D-MPNN classifier was able to match compounds with their essential *M. tuberculosis* targets. With the analysis of gene clusters and growth inhibition predictions, we believe this framework offers an innovative paradigm for modeling chemical-genetic data towards the characterization of novel drug MOAs.

## Supplementary Information

**Additional file 1: Table S3.** Classification results and optimum cutoffs for each model in 13 clusters. AUROC, and AUPRC for each cluster on the test set were measured and averaged. The Cutoffs were determined by Youden’s index, then accuracy and F1 Score were also included. **Table S4.** Classification results of ten-fold cross validation for each model in 13 clusters. The cutoffs were determined by Youden’s index from the previous validation set of the scaffold split. The point estimate of the mean and the error bound of population mean (EBM) at 95% confidence level of AUROC, AUPRC, accuracy and F1 score across 10 randomly partitioned data splits were presented. **Table S5.** Summary of hyperparameters and model parameters in all models. Hyperparameters of the models were obtained by Bayesian optimization ran for 30 epochs in 20 iterations on the scaffold split. Depth represents the number of the message passing iterations in D-MPNN or MPNN. FF layer represents the number of feed-forward layers in the models. **Figure S1.** Pairwise semantic similarity matrix of gene clusters. Clusters with higher semantic similarity have greater values between them. **Figure S2.** Kernel density estimate of Z-score for each *M. tuberculosis* gene cluster. **Figure S3.** Classification metrics for the D-MPNN with RDKit descriptors and baseline models using 10-fold cross validation. **A** The point estimate of the mean and the error bound of population mean (EBM) at 95% confidence level of AUROC in each cluster for all models. Only the values of D-MPNN with RDKit descriptors are displayed. **B** The mean and the EBM at 95% confidence level of metrics (AUROC, accuracy, AUPRC, F1) over clusters for all models.

**Additional file 2: Table S1.** Homologous genes between *M. Tuberculosis* and *E. Coli* after homology search through BLAST.

**Additional file 3: Table S2.** Annotation of the *M. Tuberculosis* genes and clusters. *M. tuberculosis* genes are represented by gene symbols and the protein RefSeq ID and COG category eggNOG.

**Additional file 4: Table S6.** Ground truth and prediction of curated *M. Tuberculosis* inhibitors.

## Data Availability

The raw data from the Johnson et al. study is publicly accessible on the web site: https://www.chemicalgenomicsoftb.com/. The scripts, datasets, and results supporting the conclusions of this article are available in the supplementary materials and in our GitHub repository: https://github.com/LCY02/Mtb_CGIP_Pred.

## Abbreviations

AUPRC: Area under the curve of the precision-recall cure
AUROC: Area under curve of receiver operating characteristic curve
BLAST: Basic Local Alignment Search Tool
BO: Bayesian optimization
BP: Biological process
CDF: Cumulative density function
CGIP: Chemical–genetic interaction profile
COG: Cluster of orthologous genes
DL: Deep learning
D-MPNN: Directed message passing neural network
EBM: The error bound for a population mean
*E.**coli*: *Escherichia* *coli*
FFN: Feed-forward network
GCN: Graph convolutional neural network
GO: Gene ontology
LFC: Log fold change
ML: Machine learning
MOA: Mechanism of action
MPNN: Message passing neural networks
*M.**tuberculosis*: *Mycobacterium* *tuberculosis*
QSAR: Quantitative structure–activity relationship
QSPR: Quantitative structure–property relationship
SMILES: Simplified molecular input line entry system
