# Supplementary material for: Deep learning-driven prediction of drug mechanism of action from large-scale chemical-genetic interaction profiles
Source: J Cheminform. 2022 Mar 12;14:12. doi: 10.1186/s13321-022-00596-6 (PMC8917716; doi:10.1186/s13321-022-00596-6)

**Supporting Information**

**Table S1** **Homologous Genes between *M. Tuberculosis* and *E. Coli* after Homology Search through BLAST.**

**Table S2** **Annotation of the *M. Tuberculosis* Genes and Clusters.** *M. tuberculosis* genes are represented by gene symbols and the protein RefSeq ID and COG category. eggNOG.

**Table S3 Classification Results and Optimum Cutoffs for Each Model in 13 Clusters.** AUROC, and AUPRC for each cluster on the test set were measured and averaged. The Cutoffs were determined by Youden’s index, then accuracy and F1 Score were also included.

| **Clusters (C)** | **C1** | **C2** | **C3** | **C4** | **C5** | **C6** | **C7** | **C8** | **C9** | **C10** | **C11** | **C12** | **C13** | **Mean** |  |
| --- | --- | --- | --- | --- | --- | --- | --- | --- | --- | --- | --- | --- | --- | --- | --- |
| **Directed Message Passing Neural Network (with RDKit Descriptors)** | | | | | | | | | | | | | | | |
| **AUROC** | 0.83 | 0.79 | 0.83 | 0.82 | 0.81 | 0.83 | 0.78 | 0.83 | 0.86 | 0.84 | 0.77 | 0.84 | 0.82 | 0.82 |  |
| **AUPRC** | 0.26 | 0.20 | 0.28 | 0.29 | 0.26 | 0.28 | 0.24 | 0.31 | 0.32 | 0.28 | 0.27 | 0.33 | 0.34 | 0.28 |  |
| **Cutoff** | 0.04 | 0.02 | 0.05 | 0.06 | 0.04 | 0.05 | 0.08 | 0.05 | 0.02 | 0.03 | 0.07 | 0.05 | 0.06 | 0.05 |  |
| **Accuracy** | 0.75 | 0.63 | 0.80 | 0.81 | 0.71 | 0.81 | 0.83 | 0.75 | 0.68 | 0.70 | 0.69 | 0.81 | 0.74 | 0.75 |  |
| **F1** | 0.16 | 0.10 | 0.19 | 0.25 | 0.16 | 0.22 | 0.22 | 0.20 | 0.16 | 0.16 | 0.21 | 0.22 | 0.27 | 0.19 |  |
| **Directed Message Passing Neural Network (without RDKit Descriptors)** | | | | | | | | | | | | | | | |
| **AUROC** | 0.81 | 0.79 | 0.82 | 0.81 | 0.80 | 0.82 | 0.78 | 0.83 | 0.84 | 0.83 | 0.76 | 0.83 | 0.81 | 0.81 |  |
| **AUPRC** | 0.21 | 0.17 | 0.25 | 0.24 | 0.24 | 0.24 | 0.20 | 0.28 | 0.28 | 0.25 | 0.24 | 0.27 | 0.32 | 0.25 |  |
| **Cutoff** | 0.05 | 0.04 | 0.05 | 0.04 | 0.06 | 0.04 | 0.08 | 0.06 | 0.03 | 0.05 | 0.10 | 0.04 | 0.07 | 0.05 |  |
| **Accuracy** | 0.80 | 0.74 | 0.78 | 0.68 | 0.76 | 0.71 | 0.82 | 0.76 | 0.69 | 0.79 | 0.78 | 0.71 | 0.73 | 0.75 |  |
| **F1** | 0.17 | 0.13 | 0.17 | 0.18 | 0.17 | 0.16 | 0.21 | 0.20 | 0.16 | 0.19 | 0.24 | 0.17 | 0.25 | 0.18 |  |
| **Message Passing Neural Network (with RDKit Descriptors)** | | | | | | | | | | | | | | | |
| **AUROC** | 0.80 | 0.78 | 0.81 | 0.80 | 0.79 | 0.81 | 0.76 | 0.82 | 0.84 | 0.82 | 0.75 | 0.82 | 0.80 | 0.80 |  |
| **AUPRC** | 0.24 | 0.15 | 0.26 | 0.26 | 0.24 | 0.25 | 0.21 | 0.27 | 0.29 | 0.26 | 0.24 | 0.29 | 0.30 | 0.25 |  |
| **Cutoff** | 0.03 | 0.03 | 0.04 | 0.04 | 0.05 | 0.03 | 0.04 | 0.04 | 0.03 | 0.03 | 0.05 | 0.04 | 0.06 | 0.04 |  |
| **Accuracy** | 0.64 | 0.68 | 0.71 | 0.60 | 0.77 | 0.60 | 0.59 | 0.72 | 0.69 | 0.68 | 0.55 | 0.77 | 0.71 | 0.67 |  |
| **F1** | 0.13 | 0.11 | 0.15 | 0.16 | 0.18 | 0.14 | 0.14 | 0.18 | 0.16 | 0.15 | 0.17 | 0.20 | 0.24 | 0.16 |  |
| **Message Passing Neural Network (without RDKit Descriptors)** | | | | | | | | | | | | | | | |
| **AUROC** | 0.80 | 0.79 | 0.82 | 0.81 | 0.79 | 0.81 | 0.78 | 0.82 | 0.85 | 0.82 | 0.76 | 0.83 | 0.79 | 0.81 |  |
| **AUPRC** | 0.23 | 0.19 | 0.28 | 0.26 | 0.26 | 0.26 | 0.22 | 0.30 | 0.31 | 0.27 | 0.26 | 0.29 | 0.33 | 0.27 |  |
| **Cutoff** | 0.03 | 0.02 | 0.03 | 0.05 | 0.05 | 0.05 | 0.05 | 0.05 | 0.03 | 0.03 | 0.05 | 0.05 | 0.05 | 0.04 |  |
| **Accuracy** | 0.74 | 0.70 | 0.75 | 0.79 | 0.77 | 0.81 | 0.75 | 0.79 | 0.73 | 0.72 | 0.62 | 0.83 | 0.75 | 0.75 |  |
| **F1** | 0.15 | 0.12 | 0.16 | 0.22 | 0.18 | 0.21 | 0.18 | 0.22 | 0.18 | 0.16 | 0.19 | 0.22 | 0.25 | 0.19 |  |
| **Feed Forward Neural Network (with RDKit Descriptors)** | | | | | | | | | | | | | | |  |
| **AUROC** | 0.78 | 0.75 | 0.79 | 0.78 | 0.76 | 0.79 | 0.73 | 0.80 | 0.83 | 0.80 | 0.74 | 0.79 | 0.79 | 0.78 |  |
| **AUPRC** | 0.23 | 0.17 | 0.26 | 0.26 | 0.24 | 0.25 | 0.22 | 0.28 | 0.30 | 0.26 | 0.26 | 0.28 | 0.31 | 0.26 |  |
| **Cutoff** | 0.05 | 0.04 | 0.06 | 0.06 | 0.06 | 0.05 | 0.06 | 0.05 | 0.04 | 0.05 | 0.08 | 0.05 | 0.08 | 0.06 |  |
| **Accuracy** | 0.72 | 0.66 | 0.79 | 0.73 | 0.69 | 0.73 | 0.71 | 0.66 | 0.67 | 0.76 | 0.64 | 0.78 | 0.76 | 0.71 |  |
| **F1** | 0.14 | 0.10 | 0.16 | 0.18 | 0.15 | 0.17 | 0.15 | 0.15 | 0.16 | 0.17 | 0.19 | 0.19 | 0.26 | 0.17 |  |
| **Feed Forward Network (with Binary Morgan Fingerprints)** | | | | | | | | | | | | | | |  |
| **AUROC** | 0.78 | 0.75 | 0.80 | 0.79 | 0.77 | 0.78 | 0.75 | 0.79 | 0.82 | 0.81 | 0.76 | 0.80 | 0.79 | 0.78 |  |
| **AUPRC** | 0.28 | 0.21 | 0.31 | 0.30 | 0.28 | 0.30 | 0.26 | 0.32 | 0.35 | 0.31 | 0.27 | 0.34 | 0.35 | 0.30 |  |
| **Cutoff** | 0.03 | 0.02 | 0.03 | 0.03 | 0.04 | 0.03 | 0.05 | 0.04 | 0.03 | 0.03 | 0.08 | 0.04 | 0.05 | 0.04 |  |
| **Accuracy** | 0.72 | 0.71 | 0.70 | 0.65 | 0.75 | 0.72 | 0.71 | 0.72 | 0.79 | 0.72 | 0.71 | 0.78 | 0.65 | 0.72 |  |
| **F1** | 0.14 | 0.11 | 0.14 | 0.17 | 0.17 | 0.16 | 0.16 | 0.17 | 0.20 | 0.15 | 0.21 | 0.19 | 0.22 | 0.17 |  |

**Table S4 Classification Results of 10-fold cross validation for Each Model in 13 Clusters.** The Cutoffs were determined by Youden’s index from the previous validation set of the scaffold split. The point estimate of the mean and the error bound of population mean (EBM) at 95% confidence level of AUROC, AUPRC, accuracy and F1 Score across 10 randomly partitioned data splits were presented.

| **Clusters (C)** | | **C1** | **C2** | **C3** | **C4** | **C5** | **C6** | **C7** | **C8** | **C9** | **C10** | **C11** | **C12** | **C13** | **Mean** |  |
| --- | --- | --- | --- | --- | --- | --- | --- | --- | --- | --- | --- | --- | --- | --- | --- | --- |
| **Directed Message Passing Neural Network (with RDKit Descriptors)** | | | | | | | | | | | | | | | | |
| **AUROC** | **Mean** | 0.80 | 0.78 | 0.80 | 0.79 | 0.78 | 0.80 | 0.75 | 0.79 | 0.82 | 0.80 | 0.74 | 0.81 | 0.80 | 0.79 |  |
|  | **EBM** | 0.01 | 0.01 | 0.01 | 0.01 | 0.01 | 0.01 | 0.01 | 0.01 | 0.01 | 0.01 | 0.01 | 0.01 | 0.01 | 0.01 |  |
| **AUPRC** | **Mean** | 0.28 | 0.23 | 0.30 | 0.29 | 0.28 | 0.30 | 0.24 | 0.31 | 0.33 | 0.29 | 0.27 | 0.32 | 0.34 | 0.29 |  |
|  | **EBM** | 0.02 | 0.03 | 0.02 | 0.02 | 0.02 | 0.03 | 0.02 | 0.03 | 0.03 | 0.03 | 0.02 | 0.03 | 0.02 | 0.02 |  |
| **Cutoff** |  | 0.04 | 0.02 | 0.05 | 0.06 | 0.04 | 0.05 | 0.08 | 0.05 | 0.02 | 0.03 | 0.07 | 0.05 | 0.06 | 0.05 |  |
| **Accuracy** | **Mean** | 0.79 | 0.69 | 0.84 | 0.83 | 0.75 | 0.84 | 0.86 | 0.79 | 0.71 | 0.75 | 0.75 | 0.84 | 0.76 | 0.78 |  |
|  | **EBM** | 0.02 | 0.02 | 0.01 | 0.01 | 0.02 | 0.01 | 0.01 | 0.01 | 0.02 | 0.02 | 0.01 | 0.01 | 0.02 | 0.01 |  |
| **F1** | **Mean** | 0.17 | 0.11 | 0.20 | 0.23 | 0.18 | 0.21 | 0.21 | 0.20 | 0.15 | 0.16 | 0.23 | 0.21 | 0.23 | 0.19 |  |
|  | **EBM** | 0.02 | 0.01 | 0.02 | 0.01 | 0.01 | 0.02 | 0.02 | 0.02 | 0.01 | 0.01 | 0.01 | 0.02 | 0.02 | 0.01 |  |
|  | **Directed Message Passing Neural Network (without RDKit Descriptors)** | | | | | | | | | | | | | | | |
| **AUROC** | **Mean** | 0.78 | 0.77 | 0.79 | 0.78 | 0.77 | 0.79 | 0.74 | 0.78 | 0.81 | 0.79 | 0.73 | 0.80 | 0.79 | 0.78 |  |
|  | **EBM** | 0.01 | 0.01 | 0.01 | 0.01 | 0.01 | 0.01 | 0.01 | 0.01 | 0.01 | 0.01 | 0.01 | 0.01 | 0.01 | 0.01 |  |
| **AUPRC** | **Mean** | 0.24 | 0.19 | 0.27 | 0.26 | 0.24 | 0.26 | 0.21 | 0.28 | 0.29 | 0.26 | 0.24 | 0.29 | 0.30 | 0.26 |  |
|  | **EBM** | 0.02 | 0.02 | 0.02 | 0.02 | 0.02 | 0.02 | 0.02 | 0.03 | 0.03 | 0.02 | 0.02 | 0.02 | 0.02 | 0.02 |  |
| **Cutoff** |  | 0.05 | 0.04 | 0.05 | 0.04 | 0.06 | 0.04 | 0.08 | 0.06 | 0.03 | 0.05 | 0.10 | 0.04 | 0.07 | 0.05 |  |
| **Accuracy** | **Mean** | 0.83 | 0.79 | 0.81 | 0.72 | 0.79 | 0.75 | 0.84 | 0.80 | 0.73 | 0.82 | 0.81 | 0.74 | 0.76 | 0.79 |  |
|  | **EBM** | 0.02 | 0.02 | 0.02 | 0.02 | 0.02 | 0.02 | 0.02 | 0.02 | 0.02 | 0.02 | 0.02 | 0.02 | 0.02 | 0.02 |  |
| **F1** | **Mean** | 0.18 | 0.13 | 0.19 | 0.17 | 0.19 | 0.16 | 0.20 | 0.19 | 0.15 | 0.19 | 0.24 | 0.16 | 0.23 | 0.18 |  |
|  | **EBM** | 0.01 | 0.01 | 0.01 | 0.01 | 0.01 | 0.01 | 0.02 | 0.01 | 0.01 | 0.01 | 0.01 | 0.01 | 0.01 | 0.01 |  |
| **Message Passing Neural Network (with RDKit Descriptors)** | | | | | | | | | | | | | | | | |
| **AUROC** | **Mean** | 0.78 | 0.77 | 0.78 | 0.78 | 0.76 | 0.79 | 0.74 | 0.78 | 0.81 | 0.79 | 0.72 | 0.80 | 0.78 | 0.77 |  |
|  | **EBM** | 0.01 | 0.01 | 0.01 | 0.01 | 0.01 | 0.01 | 0.01 | 0.01 | 0.01 | 0.01 | 0.01 | 0.01 | 0.01 | 0.01 |  |
| **AUPRC** | **Mean** | 0.25 | 0.18 | 0.27 | 0.27 | 0.25 | 0.27 | 0.22 | 0.28 | 0.31 | 0.27 | 0.25 | 0.30 | 0.31 | 0.27 |  |
|  | **EBM** | 0.01 | 0.02 | 0.02 | 0.01 | 0.02 | 0.02 | 0.02 | 0.02 | 0.02 | 0.01 | 0.02 | 0.02 | 0.02 | 0.02 |  |
| **Cutoff** |  | 0.03 | 0.03 | 0.04 | 0.04 | 0.05 | 0.03 | 0.04 | 0.04 | 0.03 | 0.03 | 0.05 | 0.04 | 0.06 | 0.04 |  |
| **Accuracy** | **Mean** | 0.68 | 0.71 | 0.74 | 0.63 | 0.78 | 0.65 | 0.63 | 0.74 | 0.71 | 0.71 | 0.56 | 0.78 | 0.72 | 0.69 |  |
|  | **EBM** | 0.01 | 0.02 | 0.01 | 0.01 | 0.01 | 0.02 | 0.01 | 0.02 | 0.02 | 0.01 | 0.01 | 0.01 | 0.01 | 0.01 |  |
| **F1** | **Mean** | 0.13 | 0.11 | 0.15 | 0.15 | 0.18 | 0.13 | 0.14 | 0.16 | 0.14 | 0.14 | 0.17 | 0.17 | 0.20 | 0.15 |  |
|  | **EBM** | 0.00 | 0.01 | 0.01 | 0.00 | 0.01 | 0.01 | 0.01 | 0.01 | 0.01 | 0.01 | 0.01 | 0.01 | 0.01 | 0.01 |  |
| **Message Passing Neural Network (without RDKit Descriptors)** | | | | | | | | | | | | | | | | |
| **AUROC** | **Mean** | 0.78 | 0.77 | 0.79 | 0.78 | 0.77 | 0.79 | 0.74 | 0.78 | 0.81 | 0.79 | 0.73 | 0.80 | 0.78 | 0.78 |  |
|  | **EBM** | 0.01 | 0.02 | 0.02 | 0.01 | 0.01 | 0.01 | 0.02 | 0.02 | 0.02 | 0.02 | 0.01 | 0.02 | 0.01 | 0.01 |  |
| **AUPRC** | **Mean** | 0.23 | 0.18 | 0.26 | 0.25 | 0.24 | 0.25 | 0.21 | 0.27 | 0.28 | 0.25 | 0.24 | 0.28 | 0.29 | 0.25 |  |
|  | **EBM** | 0.02 | 0.02 | 0.02 | 0.02 | 0.02 | 0.02 | 0.02 | 0.02 | 0.02 | 0.02 | 0.02 | 0.02 | 0.02 | 0.02 |  |
| **Cutoff** |  | 0.03 | 0.02 | 0.03 | 0.05 | 0.05 | 0.05 | 0.05 | 0.05 | 0.03 | 0.03 | 0.05 | 0.05 | 0.05 | 0.04 |  |
| **Accuracy** | **Mean** | 0.72 | 0.67 | 0.74 | 0.77 | 0.75 | 0.80 | 0.73 | 0.77 | 0.70 | 0.68 | 0.59 | 0.82 | 0.72 | 0.73 |  |
|  | **EBM** | 0.02 | 0.02 | 0.02 | 0.02 | 0.02 | 0.01 | 0.02 | 0.02 | 0.02 | 0.02 | 0.02 | 0.01 | 0.02 | 0.02 |  |
| **F1** | **Mean** | 0.14 | 0.10 | 0.15 | 0.19 | 0.17 | 0.18 | 0.16 | 0.18 | 0.14 | 0.14 | 0.18 | 0.19 | 0.21 | 0.16 |  |
|  | **EBM** | 0.01 | 0.01 | 0.01 | 0.01 | 0.01 | 0.01 | 0.01 | 0.01 | 0.01 | 0.01 | 0.01 | 0.01 | 0.01 | 0.01 |  |
|  | **Feed Forward Neural Network (with RDKit Descriptors)** | | | | | | | | | | | | | | |  |
| **AUROC** | **Mean** | 0.76 | 0.75 | 0.76 | 0.76 | 0.74 | 0.77 | 0.72 | 0.76 | 0.80 | 0.77 | 0.71 | 0.79 | 0.76 | 0.76 |  |
|  | **EBM** | 0.01 | 0.01 | 0.01 | 0.01 | 0.01 | 0.01 | 0.01 | 0.01 | 0.01 | 0.01 | 0.01 | 0.01 | 0.01 | 0.01 |  |
| **AUPRC** | **Mean** | 0.23 | 0.18 | 0.26 | 0.26 | 0.24 | 0.26 | 0.21 | 0.27 | 0.29 | 0.25 | 0.24 | 0.29 | 0.30 | 0.25 |  |
|  | **EBM** | 0.02 | 0.03 | 0.02 | 0.02 | 0.02 | 0.02 | 0.02 | 0.03 | 0.02 | 0.02 | 0.02 | 0.03 | 0.02 | 0.02 |  |
| **Cutoff** |  | 0.05 | 0.04 | 0.06 | 0.06 | 0.06 | 0.05 | 0.06 | 0.05 | 0.04 | 0.05 | 0.08 | 0.05 | 0.08 | 0.06 |  |
| **Accuracy** | **Mean** | 0.77 | 0.72 | 0.84 | 0.75 | 0.74 | 0.77 | 0.77 | 0.72 | 0.70 | 0.81 | 0.70 | 0.81 | 0.79 | 0.76 |  |
|  | **EBM** | 0.02 | 0.02 | 0.01 | 0.02 | 0.02 | 0.02 | 0.03 | 0.03 | 0.02 | 0.02 | 0.03 | 0.01 | 0.02 | 0.02 |  |
| **F1** | **Mean** | 0.14 | 0.11 | 0.19 | 0.17 | 0.16 | 0.16 | 0.16 | 0.15 | 0.14 | 0.17 | 0.20 | 0.18 | 0.23 | 0.16 |  |
|  | **EBM** | 0.01 | 0.01 | 0.02 | 0.01 | 0.02 | 0.01 | 0.02 | 0.01 | 0.01 | 0.01 | 0.01 | 0.01 | 0.01 | 0.01 |  |
| **Feed Forward Network (with Binary Morgan Fingerprints)** | | | | | | | | | | | | | | | |  |
| **AUROC** | **Mean** | 0.76 | 0.76 | 0.77 | 0.76 | 0.75 | 0.77 | 0.74 | 0.77 | 0.79 | 0.77 | 0.74 | 0.77 | 0.77 | 0.76 |  |
|  | **EBM** | 0.01 | 0.01 | 0.01 | 0.01 | 0.01 | 0.01 | 0.01 | 0.01 | 0.01 | 0.01 | 0.01 | 0.01 | 0.01 | 0.01 |  |
| **AUPRC** | **Mean** | 0.27 | 0.23 | 0.30 | 0.30 | 0.28 | 0.29 | 0.25 | 0.32 | 0.33 | 0.29 | 0.28 | 0.33 | 0.33 | 0.29 |  |
|  | **EBM** | 0.02 | 0.02 | 0.02 | 0.02 | 0.02 | 0.02 | 0.02 | 0.02 | 0.02 | 0.02 | 0.02 | 0.02 | 0.02 | 0.02 |  |
| **Cutoff** |  | 0.03 | 0.02 | 0.03 | 0.03 | 0.04 | 0.03 | 0.05 | 0.04 | 0.03 | 0.03 | 0.08 | 0.04 | 0.05 | 0.04 |  |
| **Accuracy** | **Mean** | 0.79 | 0.75 | 0.77 | 0.73 | 0.81 | 0.79 | 0.78 | 0.80 | 0.84 | 0.80 | 0.77 | 0.84 | 0.73 | 0.79 |  |
|  | **EBM** | 0.04 | 0.05 | 0.04 | 0.05 | 0.04 | 0.04 | 0.04 | 0.04 | 0.03 | 0.04 | 0.04 | 0.03 | 0.03 | 0.04 |  |
| **F1** | **Mean** | 0.16 | 0.12 | 0.16 | 0.17 | 0.19 | 0.17 | 0.18 | 0.20 | 0.21 | 0.17 | 0.23 | 0.20 | 0.21 | 0.18 |  |
|  | **EBM** | 0.02 | 0.02 | 0.02 | 0.02 | 0.02 | 0.02 | 0.02 | 0.02 | 0.03 | 0.02 | 0.01 | 0.03 | 0.02 | 0.02 |  |

**Table S5 Summary of Hyperparameters and Model Parameters in All Models.** Hyperparameters of the models were obtained by Bayesian optimization ran for 30 epochs in 20 iterations on the scaffold split. Depth represents the number of the message passing iterations in D-MPNN or MPNN. FF layer represents the number of feed-forward layers in the models.

| **Models** | **Depth** | **Hidden size** | **FF** **layer** | **Dropout** | **# Parameters** |
| --- | --- | --- | --- | --- | --- |
| **D-MPNN with RDKit** | 6 | 1100 | 1 | 0.35 | 2,746,013 |
| **D-MPNN** | 3 | 800 | 1 | 0.3 | 1,515,213 |
| **MPNN with RDKit** | 3 | 1300 | 2 | 0.4 | 5,713,513 |
| **MPNN** | 4 | 1500 | 1 | 0.25 | 4,941,013 |
| **FFN with RDKit** | - | 1400 | 2 | 0.25 | 299,613 |
| **FFN with Morgan Bits** | - | 800 | 3 | 0.35 | 2,290,413 |

**Table S6 Ground Truth and Prediction of Curated *M. Tuberculosis* Inhibitors.**

**Fig. S1 Pairwise semantic similarity matrix of gene clusters.** Clusters with higher semantic similarity have greater values between them.


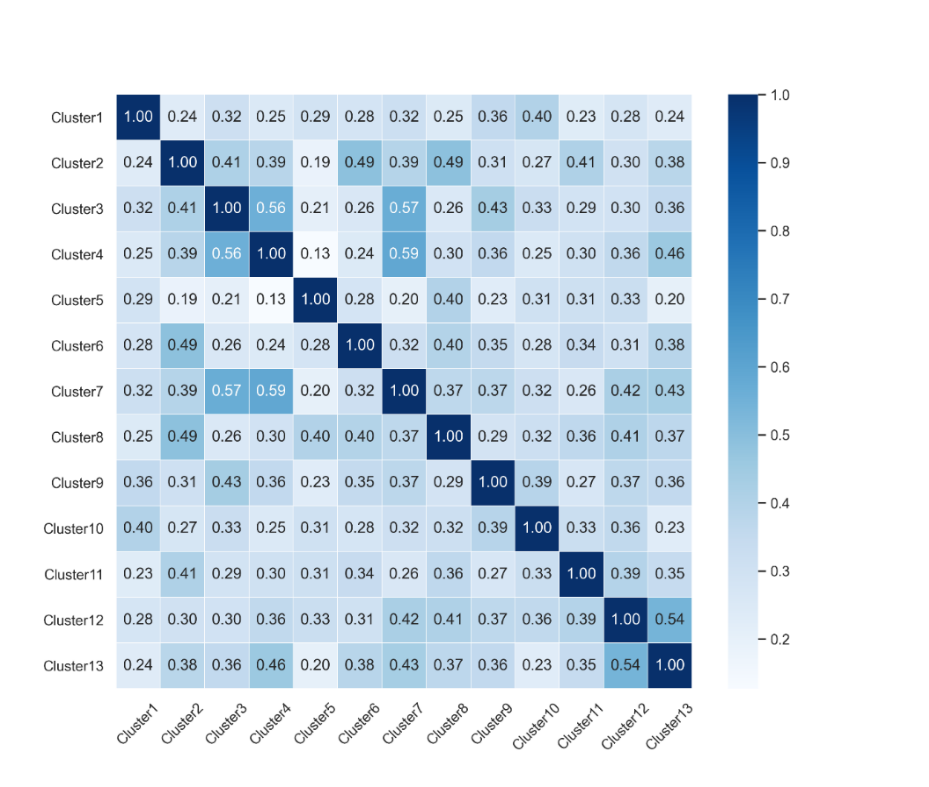


**Fig. S2** **Kernel density estimate of Z-score for each *M. tuberculosis* gene cluster.
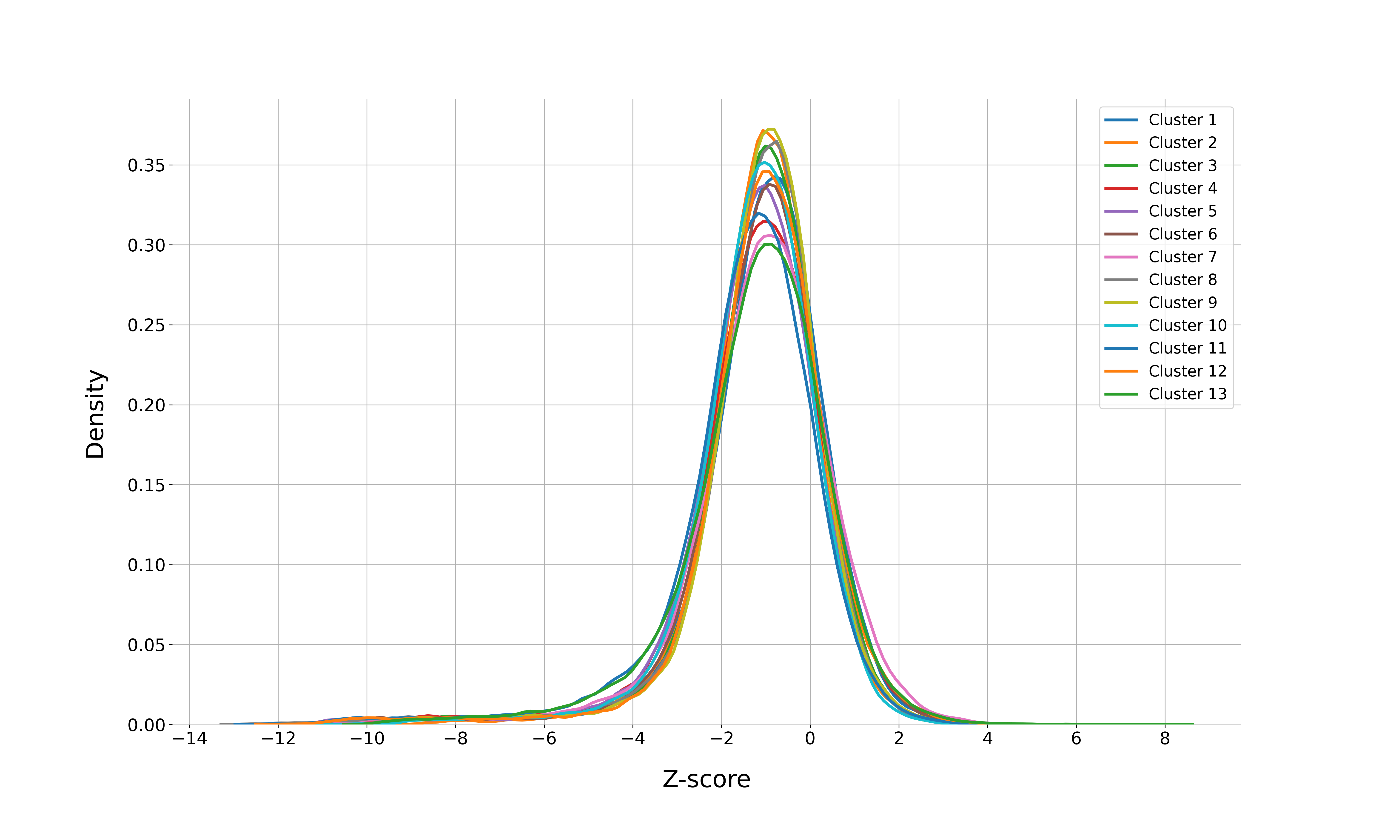
**

**Fig. S3 Classification metrics for the D-MPNN with RDKit descriptors and baseline models using 10-fold cross validation.** (A) The point estimate of the mean and the error bound of population mean (EBM) at 95% confidence level of AUROC in each cluster for all models. Only the values of D-MPNN with RDKit descriptors are displayed. (B) The mean and the EBM at 95% confidence level of metrics (AUROC, accuracy, AUPRC, F1) over clusters for all models.


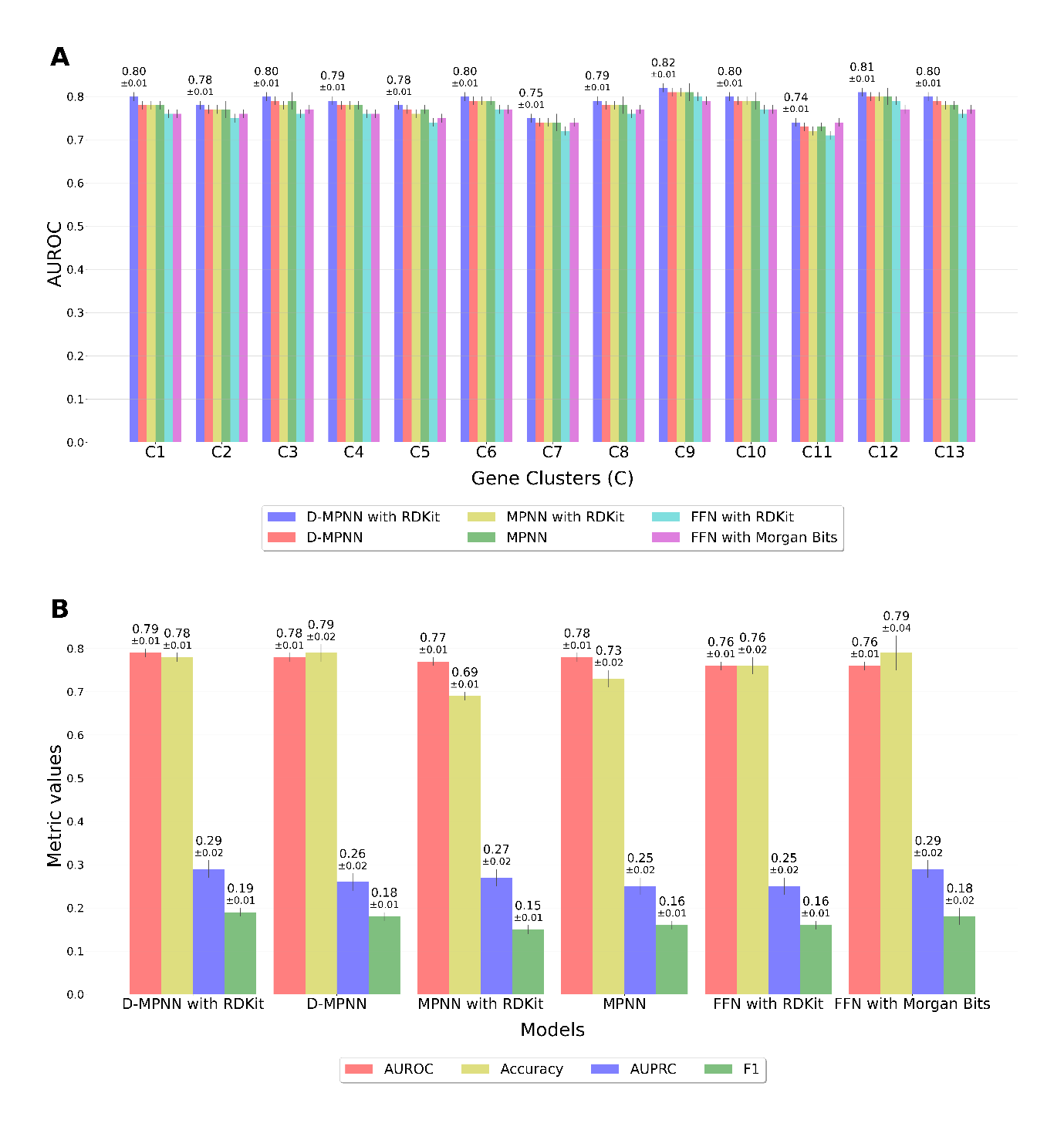

Supplement: Supplementary file 1 — Additional file 1: Table S3. Classification results and optimum cutoffs for each model in 13 clusters. AUROC, and AUPRC for each cluster on the test set were measured and averaged. The Cutoffs were determined by Youden’s index, then accuracy and F1 Score were also included. Table S4. Classification results of ten-fold cross validation for each model in 13 clusters. The cutoffs were determined by Youden’s index from the previous validation set of the scaffold split. The point estimate of the mean and the error bound of population mean (EBM) at 95% confidence level of AUROC, AUPRC, accuracy and F1 score across 10 randomly partitioned data splits were presented. Table S5. Summary of hyperparameters and model parameters in all models. Hyperparameters of the models were obtained by Bayesian optimization ran for 30 epochs in 20 iterations on the scaffold split. Depth represents the number of the message passing iterations in D-MPNN or MPNN. FF layer represents the number of feed-forward layers in the models. Figure S1. Pairwise semantic similarity matrix of gene clusters. Clusters with higher semantic similarity have greater values between them. Figure S2. Kernel density estimate of Z-score for each M. tuberculosis gene cluster. Figure S3. Classification metrics for the D-MPNN with RDKit descriptors and baseline models using 10-fold cross validation. A The point estimate of the mean and the error bound of population mean (EBM) at 95% confidence level of AUROC in each cluster for all models. Only the values of D-MPNN with RDKit descriptors are displayed. B The mean and the EBM at 95% confidence level of metrics (AUROC, accuracy, AUPRC, F1) over clusters for all models. [file 13321_2022_596_MOESM1_ESM.docx]
